# A nuclear-encoded protein, mTERF6, mediates transcription termination of *rpoA* polycistron for plastid-encoded RNA polymerase-dependent chloroplast gene expression and chloroplast development

**DOI:** 10.1038/s41598-018-30166-6

**Published:** 2018-08-09

**Authors:** Yi Zhang, Yong-Lan Cui, Xiao-Lei Zhang, Qing-Bo Yu, Xi Wang, Xin-Bo Yuan, Xue-Mei Qin, Xiao-Fang He, Chao Huang, Zhong-Nan Yang

**Affiliations:** 1 College of Life and Environmental Sciences, Shanghai Normal University, Shanghai, 200234 China; 2 Shanghai Center for Plant Stress Biology, Chinese Academy of Sciences, Shanghai, 201602 China

## Abstract

The expression of plastid genes is regulated by two types of DNA-dependent RNA polymerases, plastid-encoded RNA polymerase (PEP) and nuclear-encoded RNA polymerase (NEP). The plastid *rpoA* polycistron encodes a series of essential chloroplast ribosome subunits and a core subunit of PEP. Despite the functional importance, little is known about the regulation of *rpoA* polycistron. In this work, we show that mTERF6 directly associates with a 3′-end sequence of *rpoA* polycistron *in vitro* and *in vivo*, and that absence of mTERF6 promotes read-through transcription at this site, indicating that mTERF6 acts as a factor required for termination of plastid genes’ transcription *in vivo*. In addition, the transcriptions of some essential ribosome subunits encoded by *rpoA* polycistron and PEP-dependent plastid genes are reduced in the *mterf6* knockout mutant. RpoA, a PEP core subunit, accumulates to about 50% that of the wild type in the mutant, where early chloroplast development is impaired. Overall, our functional analyses of mTERF6 provide evidence that it is more likely a factor required for transcription termination of *rpoA* polycistron, which is essential for chloroplast gene expression and chloroplast development.

## Introduction

Chloroplasts are semi-autonomous organelles derived by endosymbiosis from a relative of present-day cyanobacteria. The plastid genome is a conserved circular double-stranded DNA of 120 to 160 kbp (154,478 bp in *Arabidopsis*)^1,2^. The *Arabidopsis* plastid genome contains 87 potential protein-coding genes, 4 rRNA genes and 37 tRNA genes^1,3^. These plastid genes are transcribed into monocistronic and polycistronic mRNAs^4^. Two types of DNA-dependent RNA polymerases, nuclear-encoded RNA polymerase (NEP) and plastid-encoded RNA polymerase (PEP), are responsible for the transcription of plastid genes^5,6^. NEP is a single-subunit enzyme that transcribes housekeeping genes, including *rpoA*, *rpoB*, *rpoC1* and *rpoC2*^7,8^. PEP possesses the major RNA polymerase activity in the chloroplast and transcribes over 80% of the plastid genes^7^. The four core subunits of PEP (α, β, β′ and β″) are encoded by the genes *rpoA*, *rpoB*, *rpoC1* and *rpoC2*, respectively, and are translated from two polycistrons, *L23-L2-S19-L22-S3-L16-L14-S8-L36-S11-rpoA* and *rpoB-rpoC1-rpoC2*^9^.

The primary transcripts produced by NEP and PEP often undergo post-transcriptional modification, including splicing, maturation, trimming of 5′ and 3′ ends and RNA editing before translation^10^. In addition, nuclear-encoded factors, such as sigma factors, pTAC and pentatricopeptide repeats (PPRs), are required for regulating the plastid gene expression mediated by both NEP and PEP^11–14^. After post-transcriptional modification, the translational events are initiated on the chloroplast ribosome^15,16^. Similar to bacteria, the chloroplast has the bacterial-type 70S ribosome, composed of rRNA and protein subunits and assembled into two multi-components of the 50S (large) and 30S (small) ribosomal subunits^17,18^. The 50S ribosomal subunit consists of 23S, 5S and 4.5 rRNA and 33 proteins, of which 24 are nuclear-encoded, whereas the 30S subunit harbors only one 16S rRNA in addition to 24 proteins, of which 12 are nuclear-encoded^19^. The rest of the chloroplast gene-encoded proteins of the translation machinery can be divided into essential and non-essential components^19^. The *L23-L2-S19-L22-S3-L16-L14-S8-L36-S11-rpoA* polycistron encodes 10 ribosome proteins (L23, L2, S19, L22, S3, L16, L14, S8, L36 and S11). Only L36 belongs to the non-essential translation machinery in both bacteria (*Escherichia coli*) and plants^19^. Despite the functional importance, the precise mechanisms underlying the transcriptional regulation of the *rpoA* polycistron remain elusive.

The mitochondrial transcription termination factor (mTERF) family was first identified to be responsible for mitochondrial transcription termination at a site adjacent to the mitochondrial 16S rRNA gene^20,21^. mTERF2 binds to the mitochondrial DNA (mtDNA) in a non-sequence-specific manner^22^. mTERF3 and mTERF2 act as positive and negative regulators in mitochondrial DNA transcription, respectively^23,24^. mTERF4 regulates the translation of mitochondrial genes by association with rRNA^25–27^. Both *mterf3* and *mterf4* knockout mice cause embryo lethality, and functional studies suggest their pivotal roles in regulating ribosome biogenesis^23–27^.

In stark contrast to mammals, in plants, the functions of mTERFs are poorly understood^28^. *Arabidopsis* contains 35 mTERF proteins, and at least 11 are predicted to be localized in the plastid^29^. SOLDAT10/mTERF1 is involved in chloroplast retrograde signaling, and absence of SOLDAT10 leads to embryo lethality^30^. *RUG2*/*BSM* (*RUGOSA2*/*BELAYA SMERT*)/*mTERF4* encodes a chloroplast protein involved in splicing group IIa introns of plastid genes^29,31^. Zm-mTERF4, the maize ortholog of the *Arabidopsis* protein BSM/RUG2, is required for accumulation of plastid ribosomes and splicing of several group II introns in the chloroplast^32^. In addition to its direct function in regulating plastid gene expression, mTERF4/COE1 is required for regulating un-processed plastid RNA-triggered plastid retrograde signaling^33^. MDA1/mTERF5 functions in chloroplast development and abiotic stress responses^30^. The *mda1* mutant exhibits a pale pigmentation^34^. *twr-1/mterf9* was identified as a yellow mutant^35^ and alters chloroplast development and tolerance to abiotic stress^36^. Although several mTERF members have been characterized in *Arabidopsis* and were emphasized to play a role in regulating organellar gene expression, whether these members also involve in the transcription termination of plastid genes is unknown.

mTERF6 is dual-targeted to both chloroplast and mitochondria in *Arabidopsis*^37^. The knockout mutant *mterf6* has an albino phenotype and is defective in the maturation of *trnI.2*^37^. By functional characterization of mTERF6, we found that mTERF6 is required for the transcription termination of plastid genes *in vivo*. The decreased RpoA protein level and PEP-dependent plastid gene transcription indicated that mTERF6-dependent transcription termination of the *rpoA* polycistron is necessary for PEP-dependent chloroplast gene expression and chloroplast development.

## Results

### The albino *mterf6* mutant turns green in sugar-containing medium

**Figure 1:**
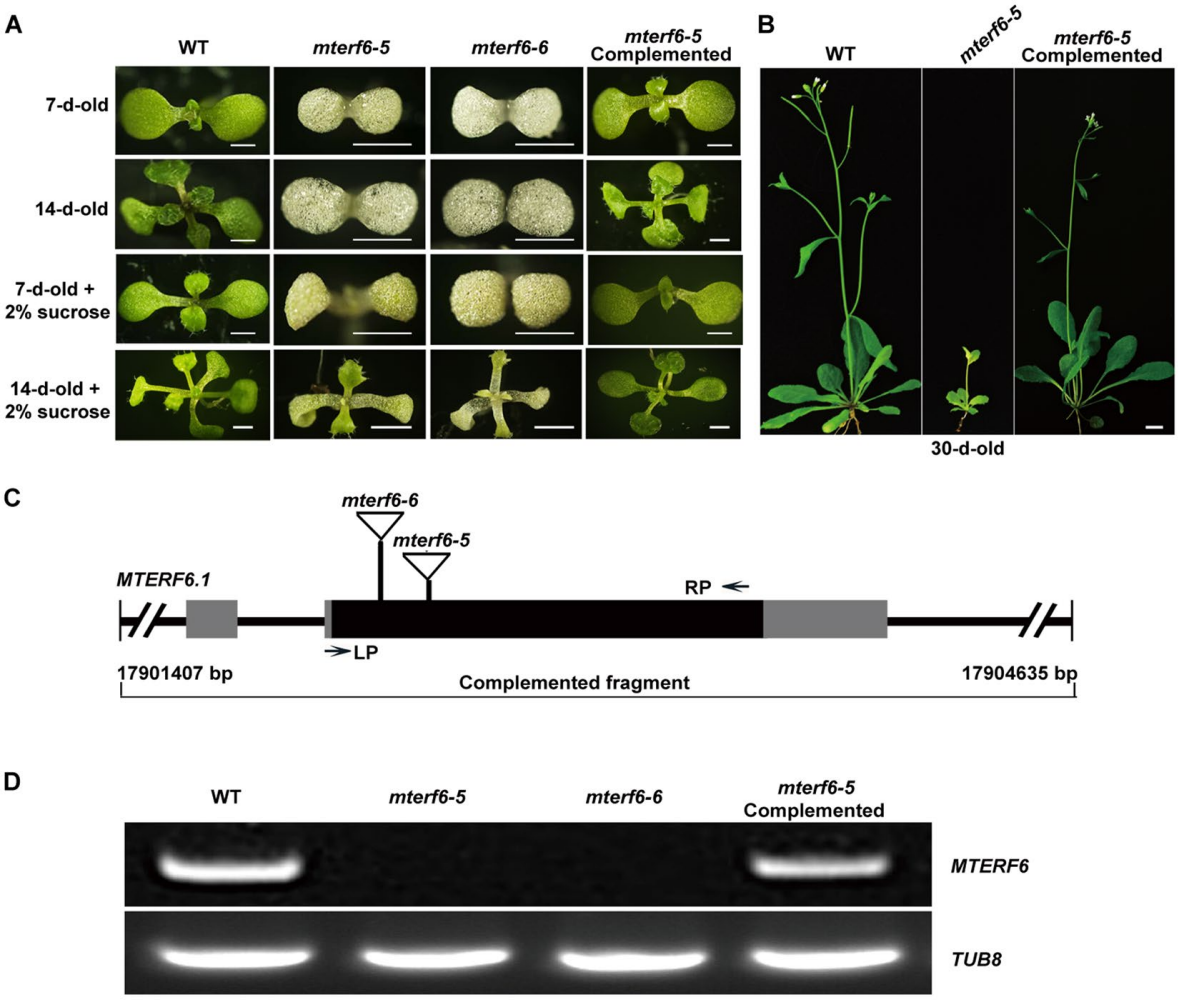
Phenotype characterization and the T-DNA insertion sites identification of the *mterf6* mutant. (**A**) Phenotype of the wild-type (WT; Columbia-0), *mterf6-5* and *mterf6-6* mutants, and *mterf6-5* complemented plants grown on MS medium with or without 2% sucrose for 7 or 14 days. Bars = 0.1 cm. (**B**) Phenotype of the WT, *mterf6-5* and *mterf6-5* complemented plant grown in soil after being grown in MS medium with 2% sucrose for 2 weeks. Bar = 1 cm. (**C**) Positions of the two T-DNA insertions in the *mterf6* mutants and the genomic fragment for complementation experiment. The black boxes, gray boxes and lines indicate the exons, untranslated regions and introns, respectively. The location of the primer pair (LP and RP) used for genotyping is shown by arrows. (**D**) *MTERF6* expression in WT, *mterf6-5* and *mterf6-6* mutants and in the complementation line.

To characterize the functions of mTERF6, we isolated its T-DNA insertion line CS16125, also named *PIGMENT DEFECTIVE 191* (*PDE191*)^38^, from the *Arabidopsis* Biological Resource Center (ABRC). The CS16125 line was renamed “*mterf6-5*” to distinguish the reported mutant lines of *mterf6-1*, *mterf6-2*, *mterf6-3* and *mterf6-4*^37^ alleles. PCR analysis with T-DNA and genome-specific primers indicated that the mutant plants are homozygous for the insertion. The progenies of the selfed heterozygote segregated with a ratio of green plants to albino plants of 233:67 [x^2^_(3:1)_ = 1.8966; P > 0.05], which suggests that this mutant is inherited as a single recessive allele. In addition, we obtained another *mterf6* allele (SALK_116335) from ABRC that was designated “*mterf6-6*”. The isolation of the genomic sequence flanking the left border of the T-DNA revealed that the T-DNAs are inserted in the first exon of *MTERF6* at positions +58-bp and +34-bp relative to the start codon in *mterf6-5* and *mterf6-6*, respectively (Fig. 1C). RT-PCR analysis with gene-specific primers revealed no *MTERF6* expression in *mterf6-5* and *mterf6-6* (Fig. 1D). Both *mterf6-5* and *mterf6-6* displayed an albino phenotype and also arrested chloroplast development at an early stage in MS medium without sucrose (Fig. 1A and Supplementary Fig. 1A,B). The mutants did not produce true leaves and were seedling lethal after being grown in soil for about 2 weeks (Fig. 1A and Supplementary Fig. 1A). When grown on 2% sucrose-containing MS medium, both *mterf6-5* and *mterf6-6* produced green true leaves at about 2 weeks (Fig. 1A and Supplementary Fig. 1A). Meanwhile, chloroplasts developed stromal thylakoids (STs). However, these STs were irregular and did not form intact grana thylakoid (GT) stacks as compared with the wild type (WT) (Supplementary Fig. 1B). After being transplanted into the soil from sucrose-containing medium (grown for <2 weeks), the plants survived, turned green and flowered (Fig. 1B). However, they grew very weakly and barely produced any seeds (Fig. 1B).

To further confirm whether the T-DNA insertion in *mterf6* was responsible for its albino phenotype, a 3229-bp genomic fragment of *AT4G38160* gene containing the 1005-bp upstream, the 1493-bp intragenic sequence (from the initial codon to the stop codon, comprising three exons and two introns) and the 731-bp downstream sequences (Fig. 1C) was cloned into the binary pCAMBIA1300 vector and then transformed into heterozygous plants (*MTERF6/mterf6-5*) via *Agrobacterium tumefaciens*-mediated transformation^39^. Among the 40 identified transgenic plants, 5 had the *mterf6-5*/*mterf6-5* background. Meanwhile, we also generated FLAG tagged complementation lines (see Methods and Supplementary Fig. 1C). These plants displayed the same phenotype as the WT (Fig. 1A,B and Supplementary Fig. 1). RT-PCR and western blot analysis revealed the *MTERF6* transcript and mTERF6 protein in the complementation line (Fig. 1D and Supplementary Fig. 1C). These data demonstrate that the mutation within *MTERF6* is responsible for the *mterf6* albino phenotype.

### *MTERF6.1* is the major transcript for mTERF6 function

**Figure 2:**
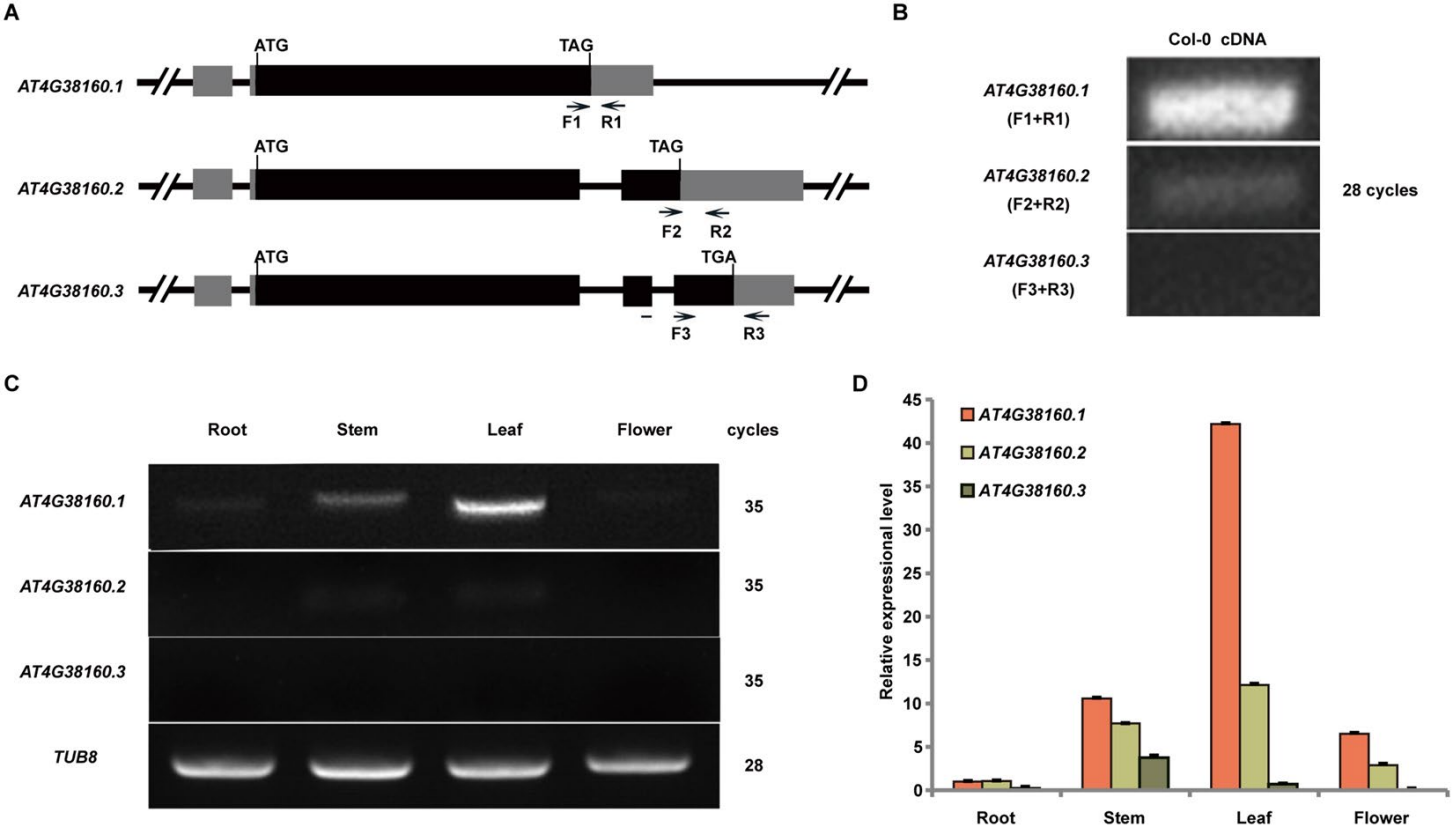
Tissue expression pattern of the *MTERF6* gene. (**A**) The sketch map of *MTERF6* and the position of the three specifically designed primers pairs (F1, R1; F2, R2; F3, R3). Black boxes show exons, gray boxes are UTRs, and black lines between boxes are introns. **(B)** The expression levels of the three transcripts in seedlings. (**C**,**D**) RT-PCR analysis (**C**) and quantitative real-time RT-PCR analysis **(D)** of *MTERF6* transcripts in root, stem, leaf, and flower. Error bars indicate standard deviations for triplicates.

According to *The Arabidopsis Information Resource* (http://www.arabidopsis.org/), *MTERF6* shows alternative splicing to produce three transcripts named *AT4G38160.1*, *AT4G38160.2* and *AT4G38160.3* (Fig. 2A). We designed three sets of primers able to specifically detect the three transcripts (Fig. 2A). Semiquantitative RT-PCR analysis revealed *AT4G38160.1* as the most highly expressed and *AT4G38160.3* weakly expressed (Fig. 2B). The expression of *MTERF6* was further detected in various tissues. Both *AT4G38160.1* and *AT4G38160.2* were highly expressed in leaves (Fig. 2C,D). *AT4G38160.3* was mainly expressed in stems and leaves (Fig. 2D). The expression pattern of the *MTERF6* with quantitative real-time RT-PCR largely agreed with that by semiquantitative RT-PCR (Fig. 2D). Overall, the expression was higher for *AT4G38160.1* than the other two transcripts in the different tissues. Furthermore, complementation assay (see Methods) demonstrated that all three genomic sequences (all containing the genomic sequence of *MTERF6.1*, Fig. 2A) of the three transcripts could complement the phenotypic defects of *mterf6-5*. Thus, *MTERF6.1* may be the major transcript for the mTERF6 function. Therefore, we investigated the function of mTERF6 based on *AT4G38160.1* (*MTERF6.1*).

The predicted products of the three transcripts of *MTERF6* are a 37.9-kD protein of 333 amino acids, a 41.3-kD protein of 363 amino acids, and a 43.1-kD protein of 378 amino acids. The structures of these proteins are similar to that of members of the mTERFs family reported in metazoans and plants^40^. SMART analysis revealed that each protein contains 7 mTERF-motif repeats (http://smart.embl-heidelberg.de/) (Supplementary Fig. 2A,B). Each motif has a length of 32 or 33 amino acids (Supplementary Fig. 2B,C). These seven motifs show high amino acid identity with each other. A proline (P) residue is highly conserved at position 8 of each of the mTERF motifs. Positions 10, 11, 15, 19, and 26 are leucine (L) or other hydrophobic amino acids, such as isoleucine (I), valine (V), or phenylalanine (F) (Supplementary Fig. 2C), which resemble that of mTERF1 in humans^41^. Previous studies of mTER1 revealed that these mTERF motifs are involved in nucleic acid binding^21^. In addition, these mTERF motifs are very conserve among plant species (Supplementary Fig. 2D).

### Transcription of PEP-dependent genes are reduced in the *mterf6-5* mutant

**Figure 3:**
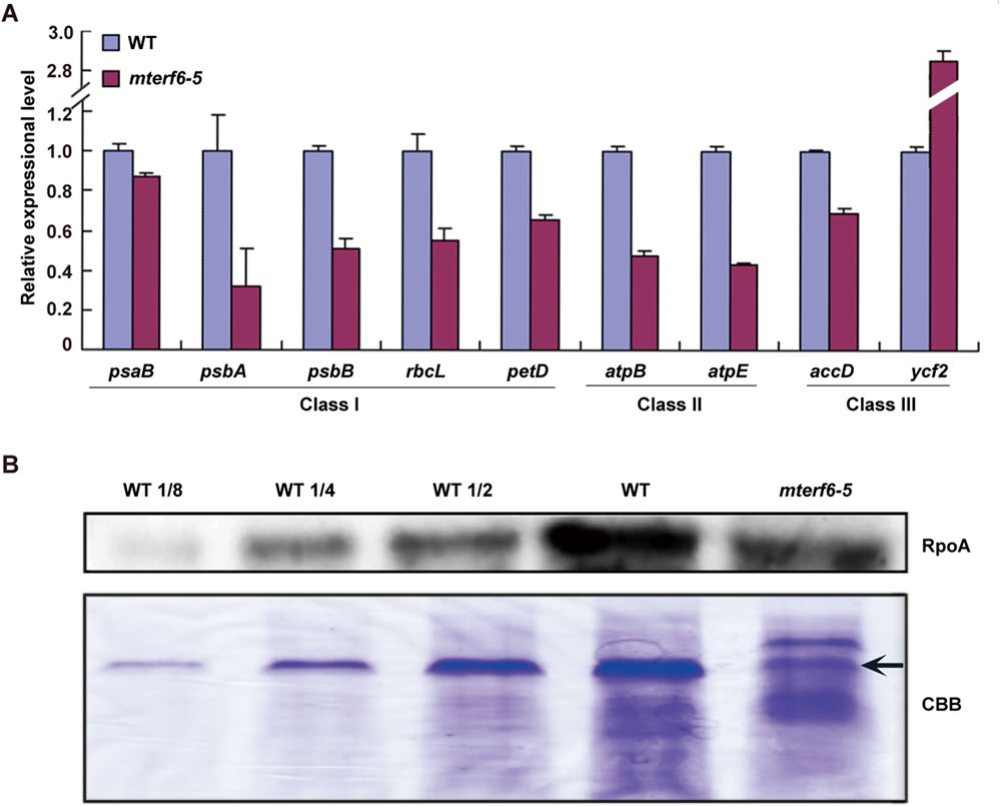
Characterization of the expression of three types of chloroplast genes and the RpoA protein accumulation in the WT and *mterf6-5* mutant. (**A**) Quantitative real-time RT-PCR analysis of the three types of chloroplast genes in 7-day-old seedlings of WT and *mterf6-5*. Error bars indicate standard deviations for triplicates. (**B**) Western blot analysis of RpoA protein accumulation in 7-day-old seedlings of WT and *mterf6-5*. Coomassie brilliant blue R-250 (CBB) staining was a loading control. Each lane contains 40 μg total protein. Lane WT 1/8, WT1/4, WT 1/2 represents that the loading amount is 1/8, 1/4, 1/2 that of the WT, respectively. The arrow shows the position of the Rubisco large subunit (RbcL).

mTERF6 belongs to one of the 35 members of the mTERF family and is localized at both mitochondria and chloroplasts^29,37^. Many plastid-localized mTERFs were found to affect chloroplast morphology/structure related to plastid gene expression^42^. PEP activity was reported critical for the leaf color phenotypes. Previous studies of a *fln2-4* mutant in our lab showed that it possesses about 30% to 40% PEP activity. The *fln2-4* mutant shows a similar albino phenotype as the *mterf6* mutant and can also turn green in sucrose-containing medium^43^. We assayed the expression of the PEP-dependent plastid genes in *mterf6-5* and WT by quantitative real-time RT-PCR. Five genes, *psaB*, *psbA*, *psbB*, *petD*, and *rbcL*, were selected as PEP-dependent genes (class I), and *ycf2* and *accD* were chosen as NEP-dependent genes (class III). The genes *atpB* and *atpE* were selected as both PEP and NEP-dependent genes (class II)^44–46^. The transcriptional levels of both the class I and II genes were downregulated compared with the WT (Fig. 3A), so PEP activity might be reduced in the *mterf6* mutant.

RpoA is one of the core subunits of PEP and is responsible for PEP activity. We used western blot analysis to examine the accumulation of the RpoA protein with a polyclonal antibody^47^ against RpoA in *mterf6-5*. RpoA level in *mterf6-5* was approximately 1/2 that in the WT (Fig. 3B). The reduced RpoA protein level in the *mterf6-5* mutant plant may further affect the PEP-dependent plastid gene expression.

### The transcription termination of the *rpoA* polycistron is defective in *mterf6*

**Figure 4:**
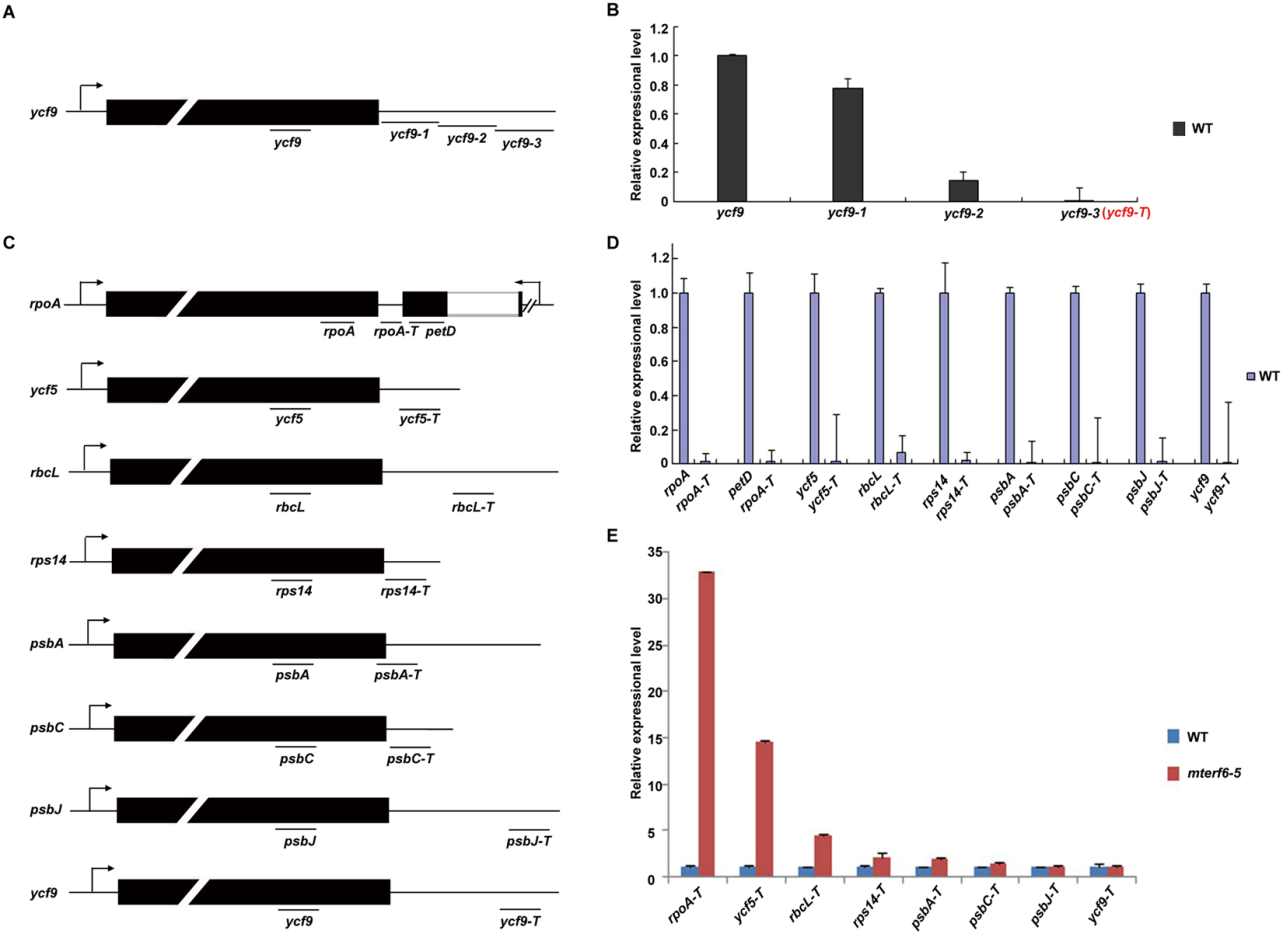
Quantitative real-time RT-PCR analysis of the read-through transcripts in the WT and *mterf6-5*. (**A**) An example of the *ycf9* gene for the primer design. The primer sets of the inside (*ycf9*) and downstream (*ycf9-1*, *ycf9-2*, *ycf9-3*) are shown in the model. “Normal” represents supposed normal transcripts of *ycf9*, and “elongated” represents supposed elongated transcripts of *ycf9*. (**B**) Quantitative real-time RT-PCR analysis of the mRNA level of *ycf9* in WT by primer sets. The primer pair of *ycf9-3* was renamed “*ycf9-T*” (red) and selected for further study. Error bars indicate standard deviations for triplicates. **(C)** Selected primer sets and their locations in their corresponding genes. (**D**) Comparison of the expression of genes with the selected primer sets by quantitative real-time RT-PCR in the WT. Error bars indicate standard deviations for triplicates. (**E**) Comparison of the expression of genes with the selected primer pairs by quantitative real-time RT-PCR in the WT and *mterf6-5*. Error bars indicate standard deviations for triplicates.

The genes *rpoA*, *rpoB*, *rpoC1* and *rpoC2* encode 4 core subunits of PEP^7^. We investigated whether mTERF6 can terminate the expression of *rpoA* (and/or *rpoB*-*rpoC1*-*rpoC2*) and some other plastid genes. Since the exact termination sites of the plastid transcripts are not clear, we designed primer sets to characterize the non-transcribed regions of the plastid genome. We used *ycf9* as an example to identify the downstream non-transcription region (Fig. 4A). The primer set (*ycf9*) inside the gene and several primer sets downstream (*ycf9-1*, *ycf9-2*, *ycf9-3*) that cover the gene interval region, were designed (Fig. 4A). Total cDNA from the WT was used as a template for quantitative RT-PCR analysis. The transcripts detected by the primer sets *ycf9-1* and *ycf9-2* did not differ in magnitude from that with the primer set *ycf9* (Fig. 4B). In contract, the primer set *ycf9-3* barely detected the transcript (Fig. 4B), which suggests that the primer set *ycf9-3* is located in the region downstream of the termination site. This primer set was renamed “*ycf9-T*” (in red, Fig. 4B) and used for further study. Using the same method, we identified 9 primer pairs located in the regions downstream of the termination sites of *rpoA*, *petD*, *ycf5*, *rbcL*, *rps14*, *psbA*, *psbC*, *psbJ* and *ycf9* (Fig. 4C,D). These primer sets were used to detect the potential transcription read-through caused by a defective mTERF6. The transcripts from the non-transcribed spacer regions of the four chloroplast genes (*rpoA*, *petD*, *ycf5*, *rbcL*) were higher in *mterf6-5* than the WT (Fig. 4E), which suggests that the read-through transcription of these genes occurred in *mterf6-5*. The fragment *rpoA-T* is located at the intergenic region of the *rpoA* and *petD* polycistrons, which are transcribed in both orientations (Fig. 4C). Because the elongated transcripts may be caused by accumulation of *rpoA* polycistronic precursor, we designed another primer pair “*rpoA-3*” that might be located near the 3′-end of the precursor (Supplementary Fig. 4A). The increase fold (*mterf6*/WT) of *rpoA-3*′ transcript is far less than the fold change (*mterf6*/WT) of *rpoA-T* transcript (Supplementary Fig. 4B), which hints the accumulated read-through transcripts are mainly from transcription elongation. Hence, mTERF6 is involved in the transcription termination of the *rpoA* polycistron.

### mTERF6 can bind to the 3′-end of the DNA sequence of *rpoA*

**Figure 5:**
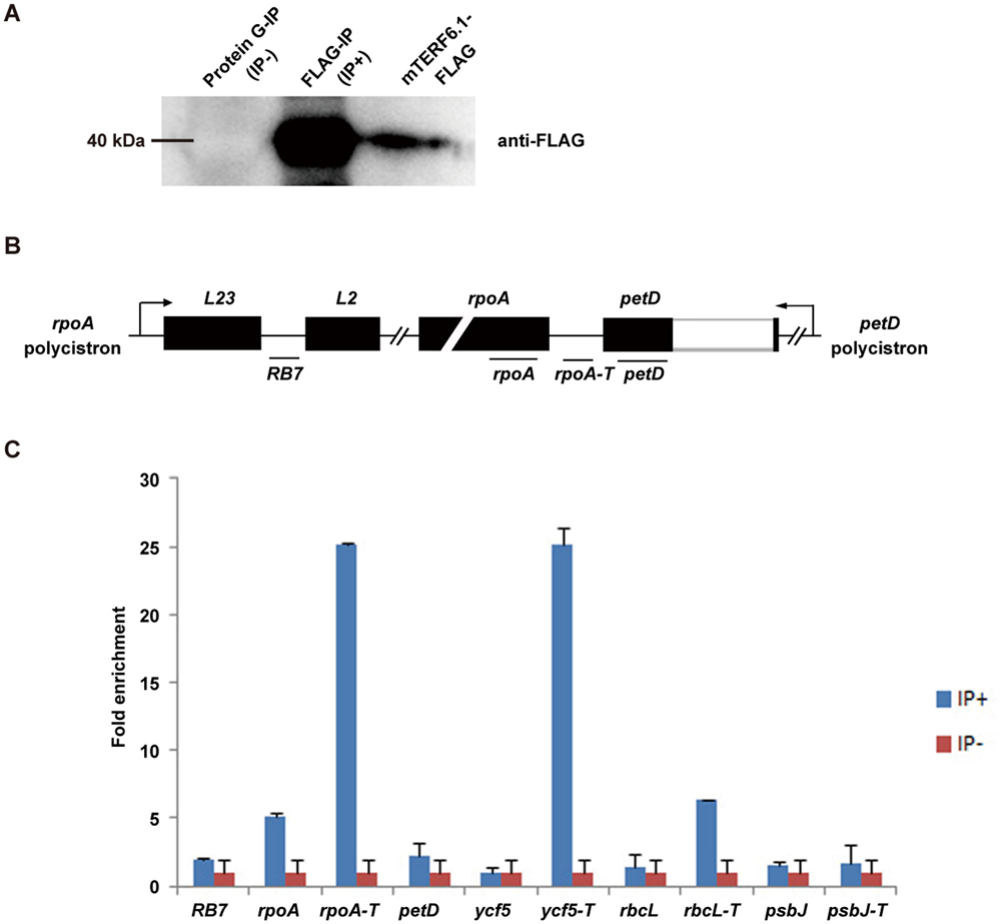
Chromatin immunoprecipitation quantitative PCR (ChIP-qPCR) analysis of the DNA binding activity of mTERF6 *in vivo*. (**A**) Immunoprecipitation and immunoblot analysis of mTERF6. The samples consisting of the total leaf protein extract from *pMTERF6::MTERF6.1-FLAG* transgenic plants (*mterf6-5* background) were immunoprecipitated with the FLAG antibody-conjugated protein G beads (IP+) or naked protein G beads (IP−) as a control. The immunoblot analysis was performed with the FLAG antibody. The lane “mTERF6.1-FLAG” is the input. (**B**) The position of the designed primer pairs for ChIP-qPCR analysis. (**C**) DNA immunoprecipitation analysis. The chloroplasts fixed with formaldehyde (FA) were extracted from the *pMTERF6::MTERF6.1-FLAG* transgenic plants, then all of the DNA was sonicated into short fragments under specific conditions and incubated with the FLAG antibody-conjugated protein G beads. With the interaction between the FLAG antibody and the mTERF6.1-FLAG protein, the associated DNA was immunoprecipitated and analyzed by quantitative real-time RT-PCR. Error bars indicate standard deviations for triplicates.

According to previously characterized mTERF homologues in mammals and green alga, their function in transcription termination relies on direct binding to mtDNA^20,48–50^. Protein structure and phylogenetic tree analyses revealed mTERF6 possesses a close relationship with these homologues (Supplementary Fig. 3A,B). To detect whether mTERF6 can also bind to the 3′-end regions of *rpoA*, *petD*, *ycf5*, and *rbcL* genes, we used chromatin immunoprecipitation (ChIP) assays as described^51^. We generated a construct of a native promoter driving the *MTERF6.1* genomic sequence (including 1005-bp upstream and 999-bp genomic sequences without stop codons) that was fused with a FLAG tag, *pMTERF6::MTERF6.1-FLAG* (see Methods). This construct could well complement the phenotypic defects of the *mterf6-5* mutant. This transgenic line was used for ChIP assay. The mTERF6.1-FLAG protein was immunoprecipitated from a total leaf protein extract of the transgenic plants by using an anti-FLAG antibody coupled with a magnetic bead-conjugated IgG (IP+). The IgG-conjugated magnetic beads were used for mock ChIP (IP−). The amount of the fusion protein in the immunoprecipitate samples was detected by western blot analysis with an anti-FLAG monoclonal antibody (Fig. 5A). A fragment, *RB7*, which is far from *rpoA-T* in the *rpoA* polycistron (Fig. 5B), was used as a negative control^47^. The protein-DNA complexes isolated from the *pMTERF6::MTERF6.1-FLAG* transgenic plants (*mterf6-5* background; Supplemantary Fig. 1C) were pulled down with the FLAG antibody and examined by ChIP quantitative PCR (ChIP-qPCR). The result showed a specific enrichment of mTERF6 at the 3′ ends of *rpoA* and the other two genes, *rbcL* and *ycf5* (Fig. 5C), which suggests that mTERF6.1 can bind to the 3′ terminus of these genes (with high affinity to *rpoA*) *in vivo*.

**Figure 6:**
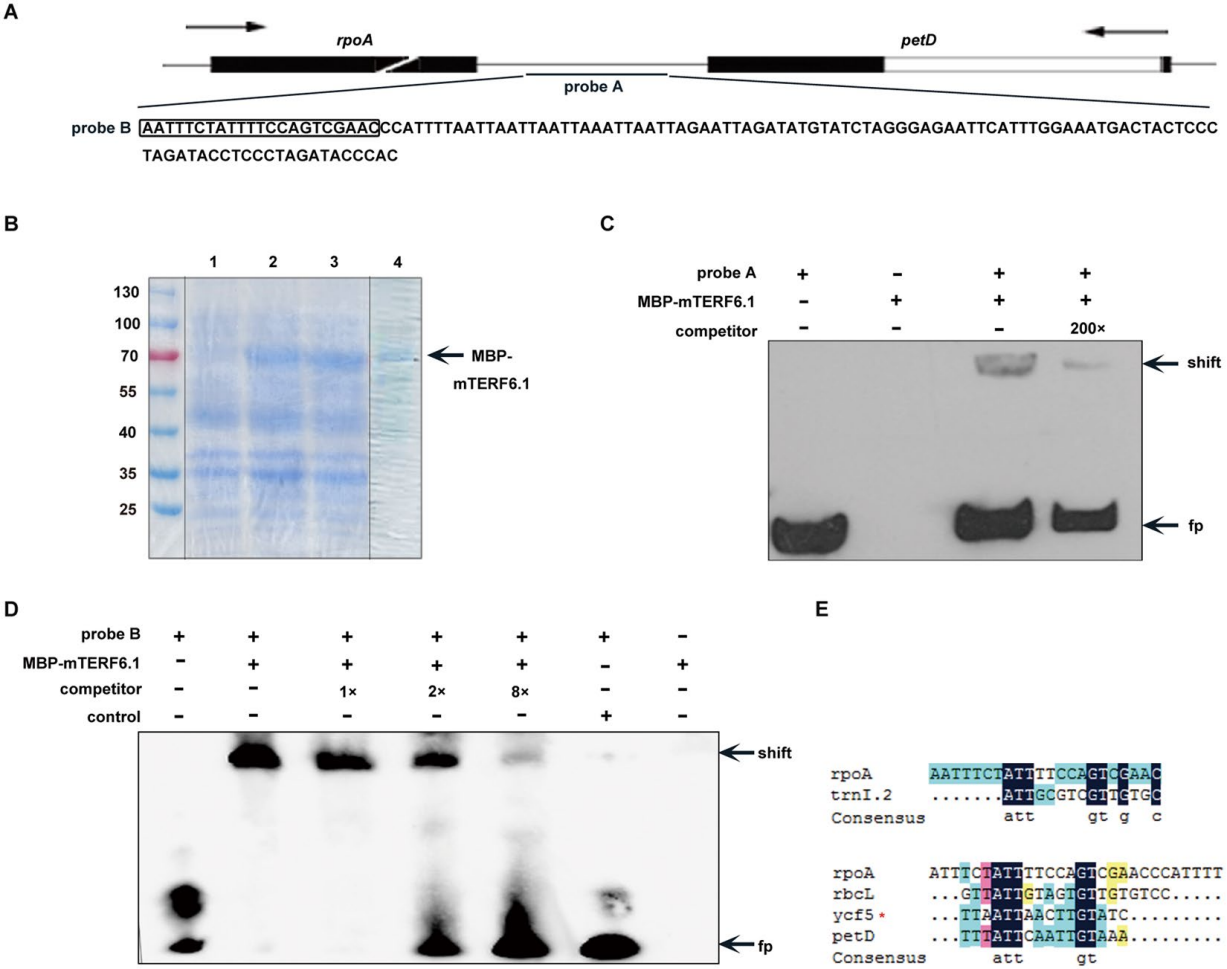
Electrophoretic mobility shift assay (EMSA) by recombinant mTERF6. (**A**) Scheme of the probes used in the EMSA assay. Black boxes, white boxes and straight lines represent the exons, introns and interval regions, respectively. The locations and sequences of probes A and B were 5′-end labeled with biotin. Probe A is a double-stranded DNA fragment produced by PCR amplification and purification. Probe B is a single strand DNA. (**B**) Expression and purification of MBP-mTERF6.1 by SDS-PAGE analysis. Lanes 1 and 2 are the total proteins from the pre-induction cultures and post-induction cultures (induced by 0.4 mM IPTG at 18 °C for 6 h), respectively; lane 3 is the soluble extract induced (by 0.4 mM IPTG at 18 °C) overnight; lane 4 is the purified recombinant MBP-mTERF6.1 protein. The arrow indicates the expected band (This is a cropped gel. Full-length gels are included in Original pictures of Supplementary Fig. 6B). (**C**) The binding of MBP-mTERF6.1 to probe A. “fp” represents free probe. (**D**) The binding of MBP-mTERF6.1 to probe B. (**E**) Comparison of the exact binding sites of *rpoA* (3′-end) and *trnI.2* and the 3′-end DNA sequences of other genes (*rbcL*, *ycf5* and *petD*). Red “*” indicates that the analyzed DNA sequences of *ycf5* are inside its downstream adjacent gene.

To further confirm whether the binding of mTERF6.1 to the 3′-end sequence of *rpoA* gene was direct, we performed electrophoretic mobility shift assay (EMSA). The interval region between *rpoA* and *petD* contains a sequence (probe B) that is similar to the binding site of the mitochondrial D-loop binding protein (mtDBP) in sea urchin (*Paracentrotus lividus*)^52^ (Fig. 6A and Supplementary Fig. 3C). A 122 bp-length fragment containing the probe B region was labeled with biotin (probe A) and used for EMSA. The mTERF6-MBP protein lacking the transit peptide was expressed and purified (Fig. 6B). A shifted band was observed when probe A was incubated with the mTERF6 protein (Fig. 6C), with no shifted signal detected in the control sample containing the purified bacterial protein expressed from the empty pMAL-c5x plasmid (Fig. 6D). When we added a 200-fold molar excess of the unlabeled 3′-end sequence of the *rpoA* gene probe A to the EMSA reaction as a competitor, the shifted signal decreased greatly (Fig. 6C), which suggests a specific binding of mTERF6 to the 3′-end sequence of *rpoA* gene probe. To further determine the exact binding sequence, we narrowed the probe to a 22-bp fragment that showed similarity to the binding sequence of the mtDBP in sea urchin (probe B) (Fig. 6A and Supplementary Fig. 3C). When probe B was incubated with mTERF6.1, a stronger shifted signal was observed (Fig. 6D). Moreover, the shifted signals became weaker when a 1-, 2-, or 8-fold competitor was added (Fig. 6D), which suggests that mTERF6.1 binds directly to the 3′-end region of *rpoA* or *petD*. No shifted signal was observed when using *RB7* as a control (Supplementary Fig. 4C). In addition, analysis of the 3′-end of *rbcL* and *ycf5* indicated that they also contain the conserved ATT(N)_5_GT sequence in their 3′-end (Fig. 6E), which suggests that mTERF6 probably recognizes conserved binding motifs in the 3′-end of their target genes. To test this, we also used the binding site *trnI.2* dsDNA as a positive control^37^. The shifted band couldn’t be competed when the conserved site ATT was mutated but still could be competed by GT mutated competitor, suggesting the ATT site is much more conserved than the GT site. (Supplementary Fig. 4C).

## Discussion

Since the first functional report of mTERF in humans^20^, mTERF factors have been demonstrated to belong to a multifunctional protein family regulating mtDNA replication, transcription termination and translation^53–55^. One of the most important functions described for the mTERF factors is the termination of mtDNA transcription at specific sites^49,50,56^. Human mTERF, also called mTERF1, is one of the best-studied mTERFs. It can regulate mtDNA transcription termination by binding two sites in mtDNA^57^. Also, the homologs of mTERF1 are involved in transcription termination in the mitochondria of other metazoans^56,58–60^ and green alga (*Chlamydomonas reinhardtii*)^50^. *Arabidopsis* contains 35 mTERF proteins with approximately 11 mTERF members localized in the chloroplasts^29^. To date, four members of the mTERF family have been functionally characterized in chloroplasts of *Arabidopsis*, and their mutants show defective plastid gene expression and chloroplast development^33,36,37,61^. However, whether these mTERFs are also involved in the transcription termination of plastid genes is unclear, although mTERF6 was reported to localize in the chloroplast and has transcription termination activity *in vitro*^37^. Here, we showed that mTERF6 is directly involved in the transcription termination of the plastid gene *rpoA* (Figs 4E, 5C, 6C, 6D) *in vivo*. Structural, evolutionary and binding-site analyses revealed that mTERF6 possesses a functional similarity in DNA binding activity with human mTERF1^37^ and the sea urchin homologue mtDBP^52^ (Supplementary Fig. 3). It seems the mTERF-mediated transcription termination may be an evolutionary-conserved mechanism that occurs in higher plants and metazoans.

A DNA region called the terminator (sequence-specific element) and transcription termination factors (DNA/RNA-bound proteins) control transcription termination. In the prokaryote, transcription is terminated by intrinsic termination and Rho-dependent termination^62–65^. In the nucleus of eukaryotes, all protein-coding RNAs and most non-coding RNAs are transcribed by RNA polymerase II (Pol II). Pol II termination depends on RNA 3′-end processing signals and termination factors^66^. In *Arabidopsis*, a nuclear-encoded factor, RHON1, was reported to terminate the transcription of the plastid gene *rbcL*^67^. It can bind to both mRNA and single-strand DNA^67^. Here, we show that mTERF6 binds with higher capacity to single strand versus double strand of 3′-end DNA of *rpoA* (Figs 5C, 6C,D and Supplementary Fig. 4C). The possible reason might be the polarity of transcription termination caused by mTERF6′s asymmetric protein structure or its polar interaction with transcription machineries (or other termination factor), which leads to mTERF6 prefers to terminate transcription from the direction of *rpoA* transcription rather than terminate from the direction of *petD* transcription^49^. Furthermore, RHON1-dependent transcription termination occurs in *Arabidopsis* but not rice (*Oryza sativa*), which suggests that the function of RHON1 in transcription termination is less conserved^67^. Protein sequence alignment showed that mTERF6 is well conserved among the plant species (Supplementary Fig. 2D). The 3′-end regions of *rpoA* from different plant species were also highly conserved (Supplementary Fig. 5). These data suggest that mTERF6 homologs in other plant species may also play a role in transcription termination.

mTERF6 was reported to bind the *trnI.2* gene directly^37^. In terms of our data, it seems that mTERF6 binding regions contain the common motif ATT(N)_5_GT (Fig. 6E). The ATT site seems to be much more conserved than GT showing by EMSA using the mutated *trnI.2* binding sites as competitors (Supplementary Fig. 4C). This result is largely in accordance with the reduced transcription termination activity of mTERF6 *in vitro* after ATTG mutation^37^. However, this motif was not identified in the 3′ ends of *rpoA* in other plant species (Supplementary Fig. 5), indicating the binding sites might undergo some variations with this protein evolution. In *rhon1*, the transcription of *rbcL* is terminated largely similar to the WT^67^. In *mterf6-5*, the amount of RT-PCR product of the *rpoA* 3′-end (*rpoA-T*) is also much less than that of its corresponding gene (Supplementary Fig. 6A,B). Thus, the transcription of *rpoA* can be terminated largely similar to that of *rbcL* in *rhon1*^67^. As in prokaryotes, the efficient transcription termination of plastid genes may also depend on RNA 3′-end processing signals besides termination factors^63,68,69^.

The *rpoA* polycistron *L23-L2-S19-L22-S3-L16-L14-S8-L36-S11-rpoA* encodes some essential ribosomal subunits and a core subunit of PEP (RpoA)^5^ which is important for plastid gene transcription and translation. mTERF6 could directly associate with the *rpoA* polycistron for transcription termination (Figs 4, 5, 6). We found the RpoA protein level downregulated in *mterf6-5* (Fig. 3B) although the *rpoA* transcripts turned out to be stable compared with the highly increased *rpoB*, *rpoC1* and *rpoC2* transcripts (Supplementary Fig. 6C). Also, most of the genes encoding essential ribosomal subunits (L23, L2, S19, L16, L14, S8 and S11) translated from the same *rpoA* polycistron were downregulated (Supplementary Fig. 6D) at early chloroplast development stage. Interestingly, *rpl2* and *rpl16* encode essential plastid ribosomal subunits, and their splicing efficiency was reduced in *mterf6-5* seedlings (Supplementary Fig. 6E). The reduced transcription of multiple transcripts of *rpoA* polycistron and *petD* (transcribed bidirectionally; Fig. 5B) indicates that mTERF6 may serve as a roadblock or a bidirectional transcription-termination factor resembling that of mtDBP in sea urchin^49^. Therefore, the loss of the transcription termination function of mTERF6 at the site (3′-end of *rpoA*; Fig. 6A) may result in transcription collision^23^ from two directions (Fig. 5B), which may ultimately reduce the total transcription level of *rpoA* polycistron (as well as *petD*) in *mterf6-5* (Fig. 3A and Supplementary Fig. 6D). Downregulated *rpl/s* transcripts at early seedling stage may lead to less amounts of essential ribosomal subunits for translation (resulting in translational defectiveness). These findings and conclusions were reinforced in the *mterf6-1* mutant as the molecular function of mTERF6 was further confirmed in seedlings of the *mterf6* weak allele (*mterf6-1*)^37^ (Supplementary Figs 7 and 8). However, the *rpl/s* transcripts are increased in soil-grown *mterf6* mutants (Supplementary Fig. 8C), which hints there may be other mechanisms in regulating transcription of these genes at soil-grown stage. For example, as these genes are regulated by NEP, NEP might be implicated in this process. The NEP activity might accumulate as the NEP-regulated genes, *accD*, *ycf2*, *rpoB*, *rpoC1* and *rpoC2*, are largely increased in soil-grown *mterf6* mutants (Supplementary Fig. 8B,C), which may compensate for the functional reduction of PEP transcribed plastid genes (Fig. 3A). Furthermore, we confirmed the *mterf6-1* displays higher transcription of PEP-dependent plastid genes than that of *mterf6-5* when grown in soil suggesting total absence of mTERF6 in the strong allele *mterf6-5* is responsible for the reduced PEP activity (Fig. 3A and Supplementary Fig. 8A,B). Notably, both *psbA* and *psbB* genes display slightly increased transcription levels in 3-week-old soil-grown *mterf6-1* mutants^37^. Different light or other growing environments may account for the differences compared with our data. RpoA is one of the core subunits of the PEP complex^5^. Both the RpoA protein level and PEP-dependent genes were downregulated in *mterf6-5* seedlings (Fig. 3). Low amounts of RpoA may lead to reduced PEP activity. In addition, the reduced translation efficiency may also lead to less RpoA protein (Supplementary Fig. 9).

The knockout mutants of mTERF6 are seedling lethal (Fig. 1), with severe chloroplast developmental defects^33^ (Supplementary Fig. 1B). A certain degree of PEP-dependent plastid transcription is critical for chloroplast development. PEP-defect mutants, such as *ptac14* and *trx z*, with very low transcription, are completely albino^70,71^. In *fln2-4*, the transcription of the PEP-dependent plastid gene is approximately 30% to 40% that of the WT and the mutant shows an albino phenotype that turns green in the sucrose-containing medium^43^. The *ecb2-2* and *ys1* mutants are also defective in the transcription of PEP-dependent plastid genes (with about 40% to 60% the transcription abundance of the WT) and have yellow leaves, which can later turn green^43^. The transcription levels of the PEP-dependent plastid genes in *mterf6-5* are similar to those of *ecb2-2* and *ys1*^43^ (Fig. 3A). However, *mterf6-5* has an albino phenotype that turns green in sucrose-containing medium (Fig. 1A), which is similar to the *fln2* phenotype^43^. These results suggest that the PEP-dependent plastid gene transcription might not be the only limiting factor for seedling growth. Seed-reserve and photosynthesis CO_2_ fixation are the two primary energy sources for seedling growth. In *fln2-4*, with a defect in chloroplast development, the seeds cannot provide sufficient energy before the biogenesis of chloroplasts^72^. The external sucrose in the medium supports its sustainable development into a functional chloroplast^72^. In *mterf6-5*, loss of mTERF6 function results in reduced translational capacity and therefore decreased photosynthesis-related protein accumulation (Supplementary Fig. 9). Thus, besides the defect in PEP activity, the reduced translational ability in chloroplasts of *mterf6* might also account for its sucrose-dependent greening phenotype.

## Materials and Methods

### Plant materials and growing conditions

Wild-type (*Columbia* ecotype, Col-0) and mutant *Arabidopsis* plants were grown on Murashige and Skoog (MS) medium containing 0.7% (w/v) agar at 22 °C with a day/night cycle of 16 h/8 h at 120 μmol m^−2^ s^−1^. The T-DNA insertion lines CS16125 and SALK_116335 were obtained from the *Arabidopsis* Biological Resource Center (ABRC; http://abrc.osu.edu/). The *mterf6-1* (GABI_152G06) mutant was kindly provided by Dr. Tatjana Kleine.

### Identification of T-DNA insertional lines and complementation of the *mterf6* mutant phenotype

For the insertion sites of the T-DNA inserted mutants, the shared LB1 primer (5′-GCCTTTTCAGAAATGGATAAATAGC-3′) was designed using the left sequence of the T-DNA. The specific primers, containing an LP and an RP, were designed by using the upstream and downstream sequences of the insertion sites, respectively. For CS16125, the primers were for LP: 5′-ACTCTGCACAAGGCATCAACC-3′; and RP: 5′-TGATGCCACATCGCTTTGAGC-3′. For SALK_116335, they were for LP: 5′- ATTGTACCCTTGTCACGCATC-3′; and RP: 5′-CGATGCTGTAGCTGATAAGCC-3′ (Supplementary Table S1). Genomic DNA (gDNA) from each mutant was extracted as the PCR template.

As for the genetic complementation experiment, the full-length 3229-bp genomic fragment (containing the sequence 1005-bp upstream of the promoter to 2224-bp downstream from the start of transcription) was amplified by KOD plus polymerase (TOYOBO; http://www.toyobo.co.jp) and was fused to the pCAMBIA1300 vector. The primer pairs were for *MTERF6-Sac-F*: 5′-gagctcGTGGGGACGTTAGGAGAGTGACCAG-3′; and *MTERF6-Sal-R*: 5′ -gtcgacTGATTACCTGCGATTATAGCCATTC-3′ (Supplementary Table S1). The recombinant DNA was introduced into the heterozygous plants (*MTERF6*/*mterf6*) by *Agrobacterium tumefaciens*-mediated transformation^39^. Then, transgenic plants were screened by PNS culture medium with 80 mg L^−1^ hygromycin and further identified with the genomic-specific primers F: 5′-ACTCTGCACAAGGCATCAACC-3′; and R: 5′-TGATGCCACATCGCTTTGAGC-3′.

### Phenotype characterization and transgenic plants generation

For phenotype characterization, plants grown on MS medium were photographed by using an anatomic microscope. The small leaf segments were collected from 7- and 14-day-old Col-0 and *mterf6-5* mutant plants and from 14-day-old complemented plants grown on MS medium (with/without 2% sucrose) under normal culture conditions. Later, a Hitachi H7500 transmission electron microscope (Hitachi High-Technologies, Tokyo) was used to observe the ultrastructure of chloroplasts^73^. For generation of transgenic plants, the 2004-bp *MTERF6.1* genomic sequence before the stop codon of *MTERF6.1* was amplified with the primer set *MTERF6-Sac-F/MTERF6.1-Sal-R* and cloned into the pCAMBIA1300 vector, following *FLAG* and 39-Nos sequences. Furthermore, the genomic sequences of *MTERF6.2* (2375 bp) and *MTERF6.3* (2495 bp) were cloned for construction by using the same method (for primer design, see Supplementary Table S1). The constructs were transformed into *MTERF6*/*mterf6-5* heterozygous plants by *Agrobacterium*-mediated transformation^39^. These transgenic plants (*pMTERF6::MTERF6-FLAG* in the *mterf6-5* mutant background) were screened and identified by using the same method as for the complementation lines mentioned above.

### RNA Isolation, cDNA Synthesis, RT-PCR, and Quantitative Real-Time RT-PCR

The procedures for the purification of total RNA, for cDNA synthesis, RT-PCR, and quantitative real-time RT-PCR were described previously^46^. The specific primers are in Supplementary Table S1.

### Western blot analysis

Total protein from a 7-day-old seedling was extracted as described^74^. The anti-RpoA polyclonal antibody was raised against a synthetic peptide of RpoA by the GL Biochem company (http://www.glbiochem.com)^47^.

### Protein Purification and Electrophoresis obility Shift Assays (EMSA)

To obtain the mTERF6 protein *in vitro*, cDNA of mTERF6 without the region of transit peptide, was cloned into the pMAL-c5X vector in-frame with the maltose-binding protein (*MBP*) gene. Nde I and BamH I sites were introduced into forward and reverse primers, respectively (see Supplementary Table S1). The expression and purification of the recombinant MBP-mTERF6 involved the pMAL Protein Fusion and Purification System (New England Biolabs). Various pairs of primers covering several sites of interest of chloroplast DNA (cpDNA) were labeled with biotin at their 5′ ends (Supplementary Table S1). The PCR products were amplified as probes by using ExTaq polymerase (Takara), and the unlabeled products were used as competitors. The double-stranded DNA probes were purified and extracted by using an Ultra-Sep Gel Extraction Kit (150) (OMEGA D2510-01). The procedures for the EMSA are available online (Light Shift Chemiluminescent EMSA Kit, Thermo Scientific; http://www.thermoscientific.com). Each 20-μL binding reaction, containing 2 μL binding buffer, 5 mM MgCl_2_, 1 mM EDTA, 2.5% glycerol, 0.05% NP-40, 50 ng/μL poly (dI-dC), 20 fmal probe and 0.5 μg recombinant protein, was performed and incubated at room temperature for 20 min.

### Chromatin immunoprecipitation (ChIP) experiments

The complemented T2 transgenic plants (*pMTERF6::MTERF6.1-FLAG* in the *mterf6-5*/*mterf6-5* background) were identified and used as material for ChIP experiments. The ChIP assay was performed as previously described^51^. Briefly, 3 to 5 g young leaves harvested from 3-week-old plants planted in soil were immersed in ~100 mL of ice-cold TBS (20 mM Tris, pH 7.6, and 200 mM NaCl) supplemented a final concentration of 0.01% (v/v) Silwet L-77 and 1% (v/v) formaldehyde. Leaves were fixed by vacuum-infiltration and washed with fresh TBS solution containing 0.3 M glycine at 4 °C. Leaves were ground in liquid nitrogen. The powder was resuspended in 3 mL of lysis buffer (50 mM Hepes-KOH, pH 7.5, 140 mM NaCl, 1 mM EDTA, 1% Triton X-100, 0.1% sodium deoxycholate, protease inhibitor cocktails, and 10% glycerol) and homogenized by vortexing. The resultant slurry was filtered through two layers of Miracloth, then the crude extracts in the filtrate were sonicated on ice with a sonifier Bioruptor (UCD-200, Diagenode). The sample was centrifuged at 15000 g for 20 min at 4 °C. The supernatant was immunoprecipitated using antibody (anti-FLAG)-coupled magnetic protein G beads (Life Technologies) (IP+) and using protein G beads (IP−) as a control. The DNA was isolated from immunoprecipitated products and used for ChIP-qPCR analysis.

### Accession numbers

Sequence data from this study can be found in the *Arabidopsis* Genome Initiative or GenBank/EMBL databases under the following accession numbers: mTERF6 (AT4G38160), RpoA (ATCG00740), PetD (ATCG00730), RbcL (ATCG00490), Ycf5 (ATCG01040), TUB8 (AT5G23860), L23 (ATCG01300), L2 (ATCG00830), S19 (ATCG00820), L22 (ATCG00810), S3 (ATCG00800), L16 (ATCG00790), L14 (ATCG00780), S8 (ATCG00770), L36 (ATCG00760), S11 (ATCG00750), PsaB (ATCG00340), PsbA (ATCG00020), PsbB (ATCG00680), Ycf2 (ATCG00860) and AccD (ATCG00500), AtpB (ATCG00480), AtpE (ATCG00470), Rps14 (ATCG00330), PsbC (ATCG00280), PsbJ (ATCG00550), Ycf9 (ATCG00300), RpoB (ATCG00190), RpoC1 (ATCG00180), RpoC2 (ATCG00170) TrnI.2 (ATCG00930).

## Electronic supplementary material

Supplementary Information
