# Supplementary material for: A nuclear-encoded protein, mTERF6, mediates transcription termination of rpoA polycistron for plastid-encoded RNA polymerase-dependent chloroplast gene expression and chloroplast development
Source: Sci Rep. 2018 Aug 9;8:11929. doi: 10.1038/s41598-018-30166-6 (PMC6085346; doi:10.1038/s41598-018-30166-6)
Supplement: Supplementary file 1 — Supplementary Information [file 41598_2018_30166_MOESM1_ESM.doc]

Supplementary Information for

**A nuclear-encoded protein, mTERF6,** **mediates transcription termination of *rpoA* polycistron for plastid-encoded RNA polymerase-dependent chloroplast gene expression and chloroplast development**

Yi Zhang^1,2,3^, Yong-Lan Cui^1,3^, Xiao-Lei Zhang^1^, Qing-Bo Yu^1^, Xi Wang^1^, Xin-Bo Yuan^1^, Xue-Mei Qin^1^, Xiao-Fang He^1^, Chao Huang^1^, Zhong-Nan Yang^1,*^

^1^College of Life and Environmental Sciences, Shanghai Normal University, Shanghai, 200234, China, ^2^Shanghai Center for Plant Stress Biology, Chinese Academy of Sciences, Shanghai, 201602, China, ^3^ These authors contributed equally to this work

*Author for correspondence: Zhong-Nan Yang

Tel: +86-21-64324650

E-mail: [znyang@shnu.edu.cn](mailto:znyang@shnu.edu.cn)

**
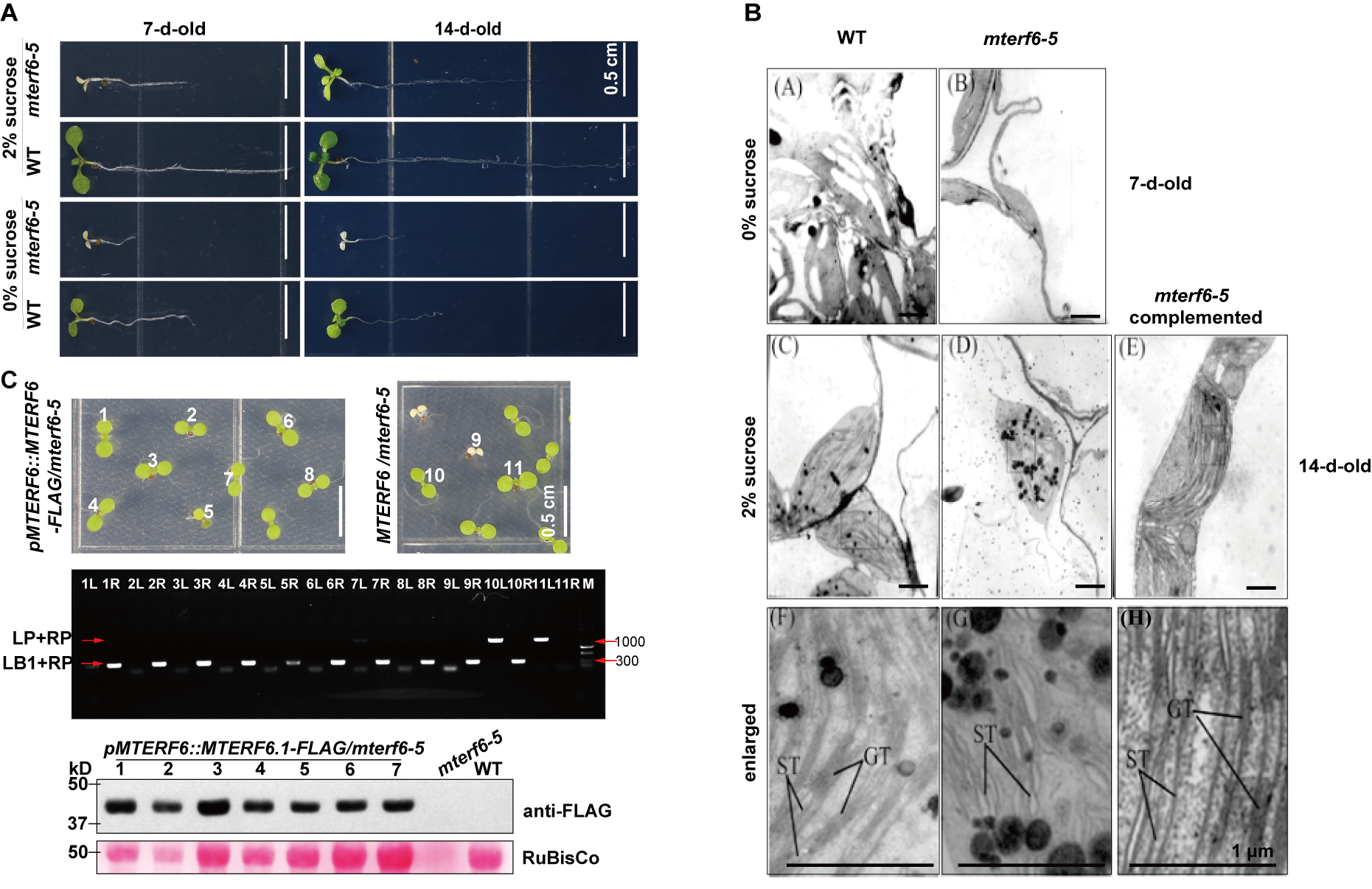
**

**Supplementary Figure 1.** Whole seedling phenotype and chloroplast ultrastructure of 7- and 14-day-old wild-type (WT), *mterf6-5* and *mterf6-5* complemented plants.

**(A)** WT and *mterf6-5* plants grown on MS medium supplemented with 0% or 2% sucrose for 7 d and 14 d, respectively. Bars = 0.5 cm.

**(B)** Ultrastructure of chloroplasts from leaves of WT, *mterf6-5*, and *mterf6-5*complemented plant by transmission electron microscopy (TEM). Choloroplast morphology of 7-d-old WT (A) and *mterf6-5* (B) grown on MS medium without sucrose, 14-d-old WT (C), *mterf6-5* (D) and complemented plant (E) grown on 2% sucrose containing MS medium. (F), (G) and (H) show the partial enlarged figures of (C), (D) and (E), respectively. GT, Grana thylakoid stacks; ST, stromal thylakoids. All bars = 1 μm.

**(C)** Identification of complementation lines of *mterf6-5* mutant. Total 11 *pMTERF6::MTERF6.1-FLAG/mterf6-5* complementation lines were identified from 70 T0 transformants (transformed *pMTERF6::MTERF6.1-FLAG* into *MTERF6/mterf6-5* heterozygotes) (data not shown). Both progenies of T1 *pMTERF6::MTERF6.1-FLAG/mterf6-5* complementation lines (No. 1 to 8) and progenies of *MTERF6/mterf6-5* heterozygous lines (No. 9, 10 and 11) were confirmed by PCR using genomic DNA as template (top panel). Primers LB1, LP and RP (Supplementary Table 1) were used to confirm the T-DNA insertion in the *mterf6-5* background (middle panel). LB1: Forward primer on left boder of T-DNA (Supplementary Table 1). L (Left) and R (Right) indicate primer pair LP + RP and LB1 + RP, respectively. The complemented plants were identified by western blot using anti-FLAG antibody (bottom panel). Total proteins were extracted from seedlings. Lane 1 to lane 7 represents total proteins extracted from 7 independent lines (top left panel). Total proteins from the *mterf6-5* mutant plants and a WT plant were used as the controls. The Rubisco large unit (RuBisCo), stained by Ponceau S staining solution, was denoted as the loading control. Bars = 0.5 cm.


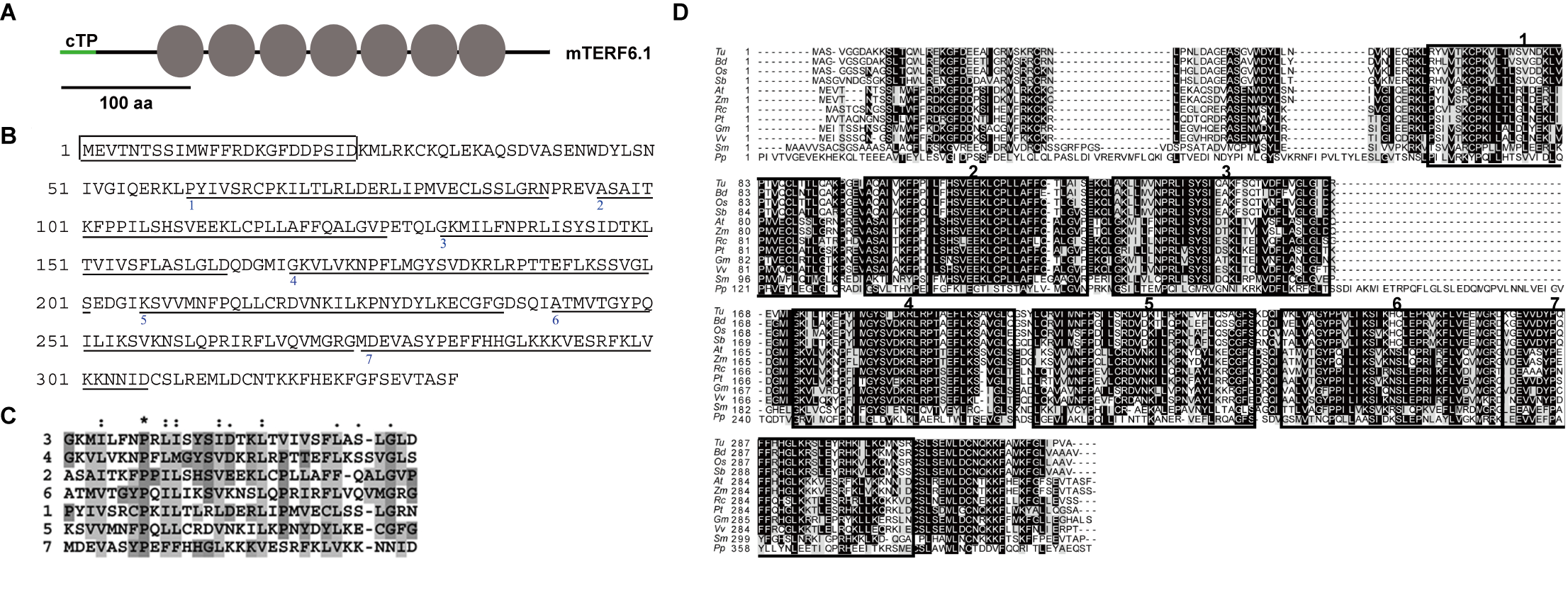


**Supplementary Figure 2.** Protein structure and sequence analyses of mTERF6.1.

**(A)** Protein structure of MTERF6.1. Each ellipse represents an “Mterf” motif. The green fragment represents a predicted N-terminal chloroplast transit peptide (cTP).

**(B)** Amino sequence of the MTERF6 protein. The leading peptide sequence (24 amino acids) identified by TargetP is framed, and the motifs identified by Pfam are underlined and noted by the numbers “1” to “7”.

**(C)** Comparison of the amino acid sequences of the 7 motifs. The similarities of the amino acid sequences are labeled by “★”, “:”, and “.”, which represent the highest, higher and high similarity, respectively.

**(D)** Protein sequence alignment among the *Arabidopsis thaliana* (*At*) mTERF6.1 and its homologs from *Triticum urartu* (*Tu*), *Brachypodium distachyon* (*Bd*), *Oryza sativa* (*Os*), *Sorghum bicolor* (*Sb*), *Zea mays* (*Zm*), *Ricinus communis* (*Rc*), *Populus trichocarpa* (*Pt*), *Glycine max* (*Gm*), *Vitis vinifera* (*Vv*), *Selaginella moellendorffii* (*Sm*), and *Physcomitrella patens* (*Pp*). The square rectangular boxes denote the 7 “Mterf” motifs of the mTERF6.1 protein.


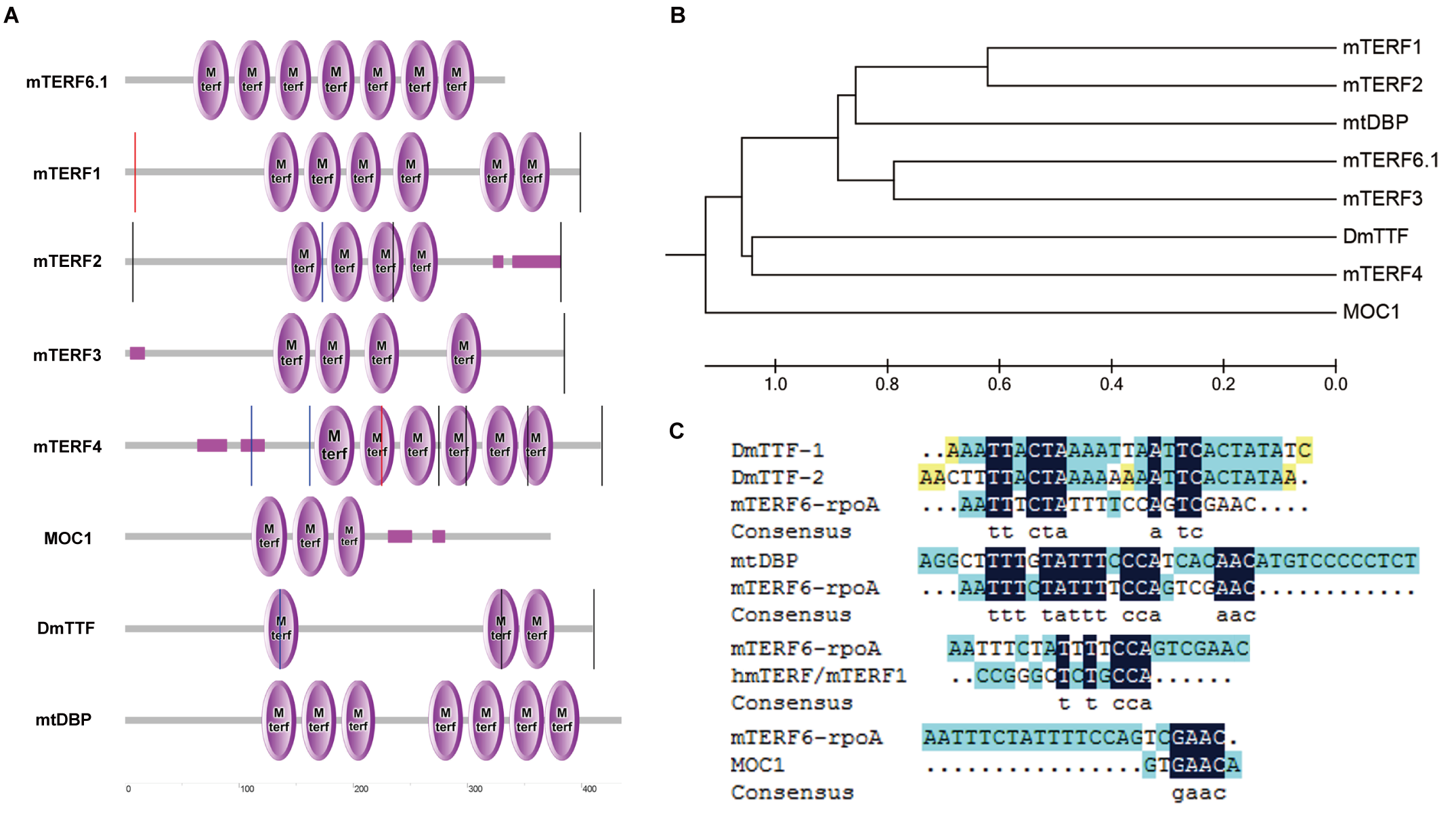


**Supplementary Figure 3.** Structural phylogenetic tree and binding sites analyses of the mTERF6 homologues.

**(A)** Protein structure of the mTERF6, mTERF6 homologues in humans and other homologues possessing transcription termination activity in green alga *Chlamydomonas reinhardtii*, *Drosophila*, and the sea urchin *Paracentrotus lividus* performed by the SMART program (<http://smart.embl-heidelberg.de/>).

**(B** and **C)** A phylogenetic tree **(B)** and a binding site comparison **(C)** of DmTTF, mtDBP, hmTERF, and MOC1 with mTERF6. The phylogenetic tree was performed by using MEGA5 software. The binding sites were acquired from published papers^48,50,58,75^.


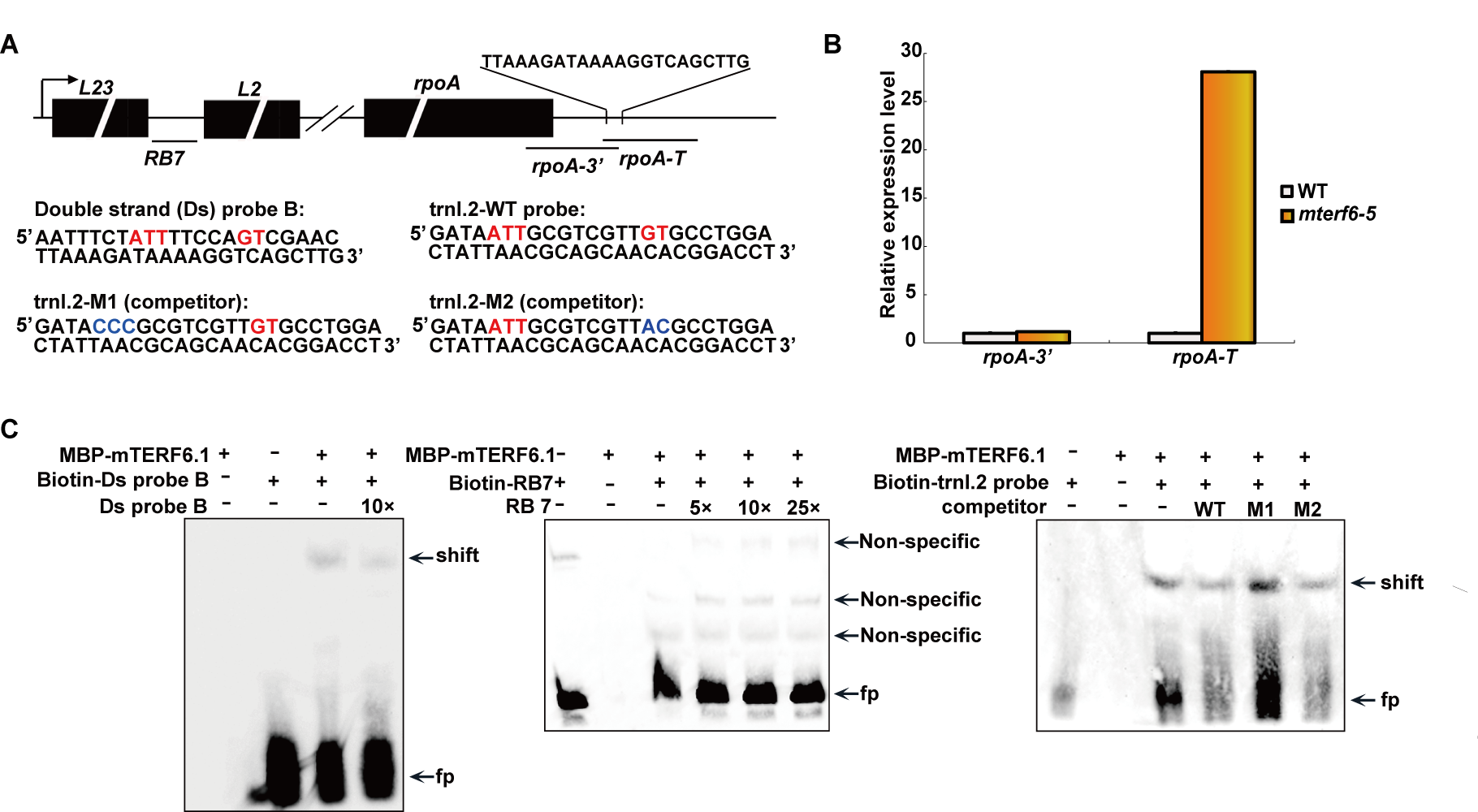


**Supplementary Figure 4.** Comparison of the *rpoA* 3’ -end transcripts in WT and *mterf6-5* and the controls of the competition experiments by using EMSA assay.

**(A)** Shematic representation of the location of the primer pairs and the DNA sequence of probes. Black boxes, white boxes and straight lines represent the exons, introns and interval regions, respectively (top panel). The proposed conserve sequences were labeled as red. Blue labeled sequences indicate the mutation sites (bottom panel). The sequences of probes for EMSA were 5’-end labeled with biotin. The competitors are non-biotin labeled DNA fragments.

**(B)** Quantitative real-time RT-PCR analysis of the *rpoA-3’* and *rpoA-T* transcripts in WT and *mterf6-5*.

**(C)** EMSA of the binding of mTERF6 to dsDNA probe B, *trnI.2* and not to RB7 probe. “fp” represents free probe. “5×”, “10×”, “25×” indicates 5-, 10- or 8-fold competitor, respectively. “WT”, “M1” and “M2” denote 10-fold competitor of trnI.2-WT, trnI.2-M1 and trnI.2-M2, respectively.


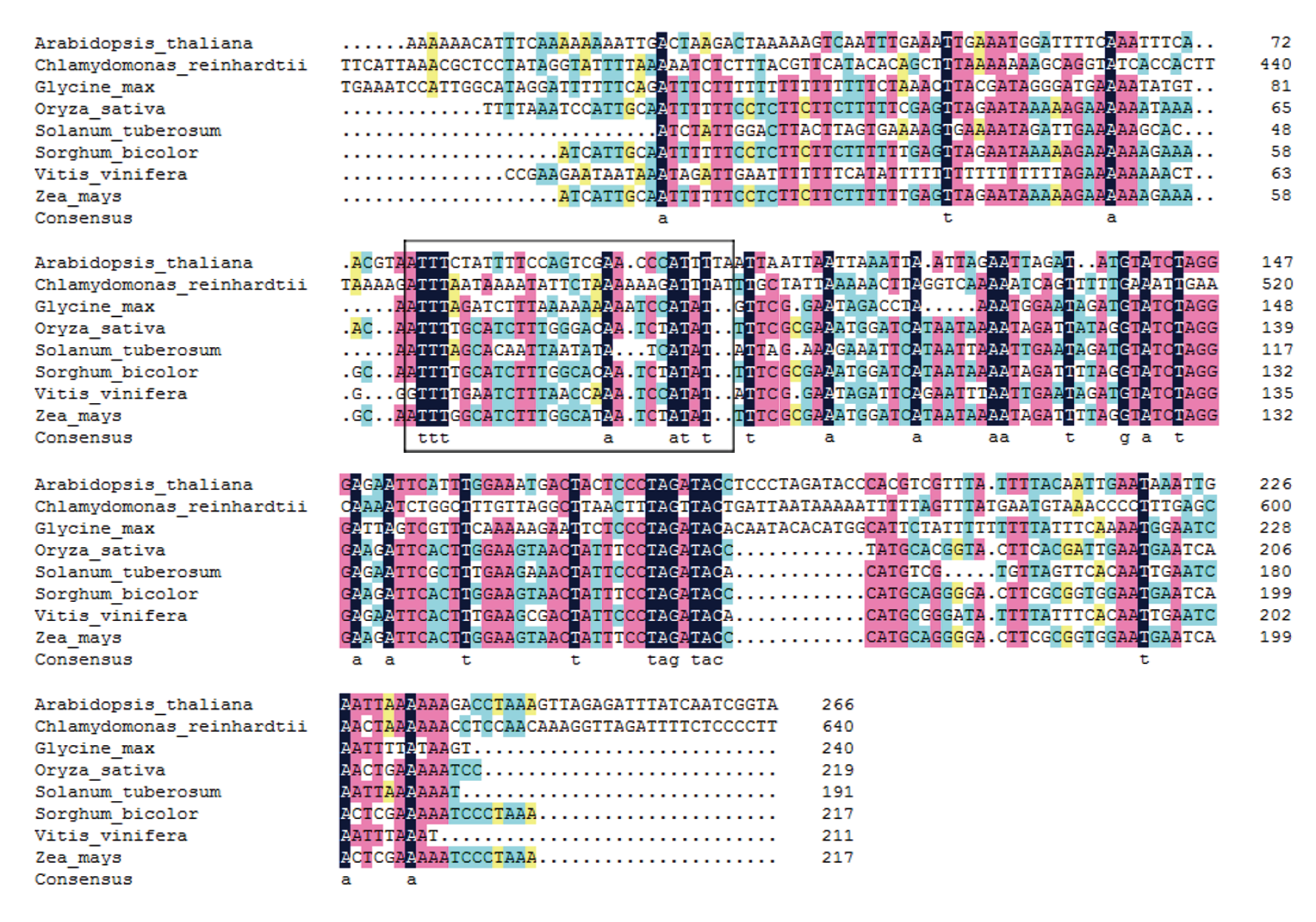


**Supplementary Figure 5.** DNA sequence alignment of the *rpoA* 3’-end region among the plant species, including *Arabidopsis thaliana*, *Chlamydomonas reinhardtii*, Glycine max, *Oryza sativa*, *Solanum tuberosum*, *Sorghum bicolor*, *Vitis vinifera* and *Zea mays*.

The square rectangular box denotes the 22-bp binding region of mTERF6. The DNA sequence information was collected from the chloroplast DB (http://chloroplast.cbio.psu.edu/organism.cgi).


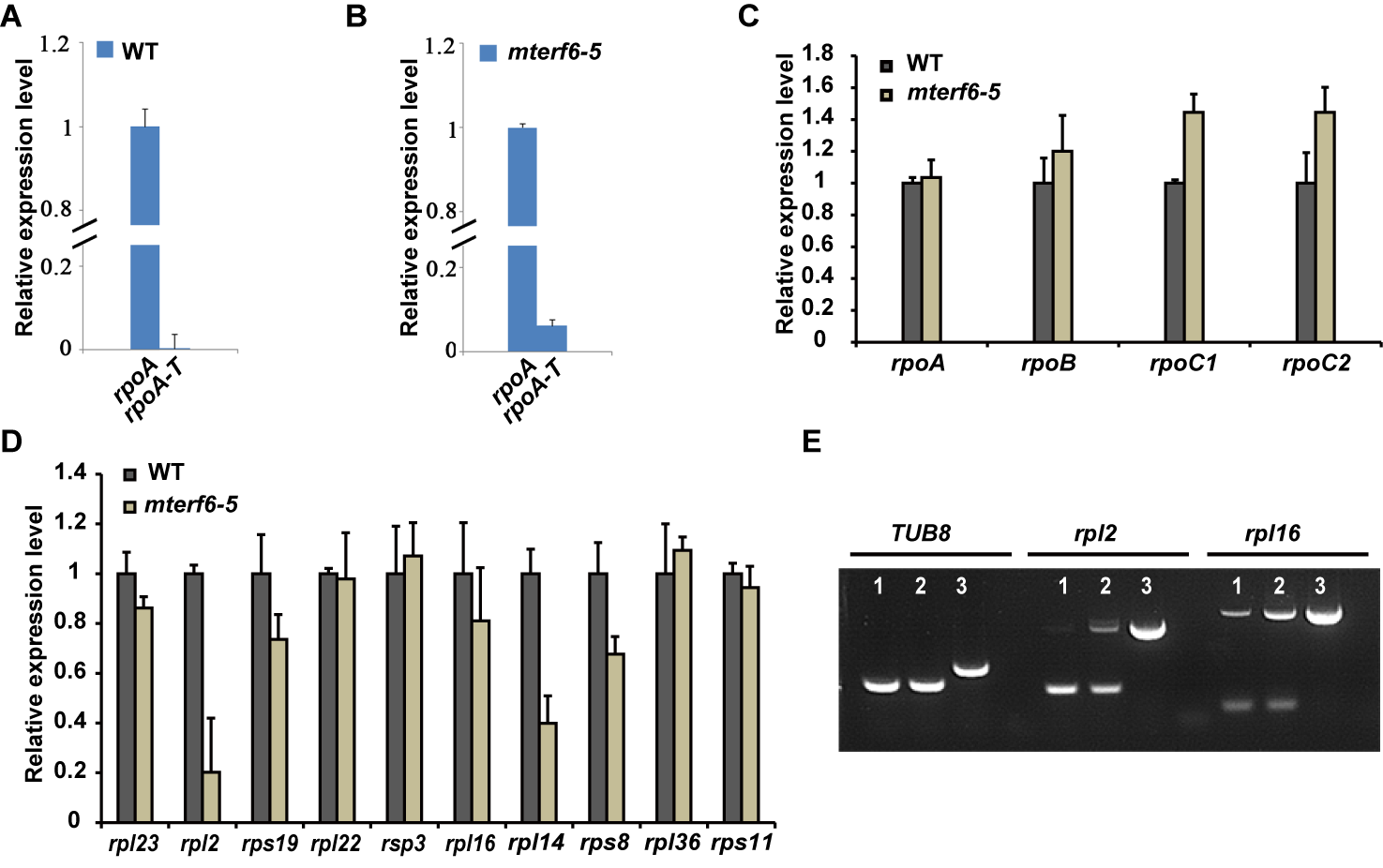


**Supplementary Figure 6.** Characterization of the transcription level and processing of the *L23-L2-S19-L22-S3-L16-L14-S8-L36-S11-rpoA* polycistron.

**(A)** A comparison of the content of the read-through transcript and total *rpoA* transcript in WT.

**(B)** A comparison of the content of the read-through transcript and total *rpoA* transcript in the *mterf6-5* mutant.

**(C)** Quantitative real-time RT-PCR analysis of the total *rpoA* transcript in WT and *mterf6-5.* The gene expression of *rpoB*, *rpoC1* and *rpoC2* (encoding β, β′ and β″ subunits of PEP) from another *rpoB*-*rpoC1*-*rpoC2* polycitron was a control.

**(D)** Quantitative real-time RT-PCR analysis of the total transcripts of *rpl23*, *rpl2*, *rps19*, *rpl22*, *rps3*, *rpl16*, *rpl14*, *rps8*, *rpl36* and *rps11* in WT and *mterf6-5*.

**(E)** Semiquantitative RT-PCR analysis of the splicing of the *rpoA* polycistronic RNA by specifically designed primer pairs of *rpl2* and *rpl16* (Supplementary Table S1). Lanes 1, 2, and 3 denote the template of the WT cDNA, the *mterf6-5* cDNA, and the WT genomic DNA, respectively.

Note: Error bars indicate standard deviations for triplicates. Total RNA was extracted from 1-week-old seedlings of WT and *mterf6-5* grown on MS medium without sucrose and reverse-transcribed. Primers for detection multiple transcripts were designed according to a previously published paper^44^.


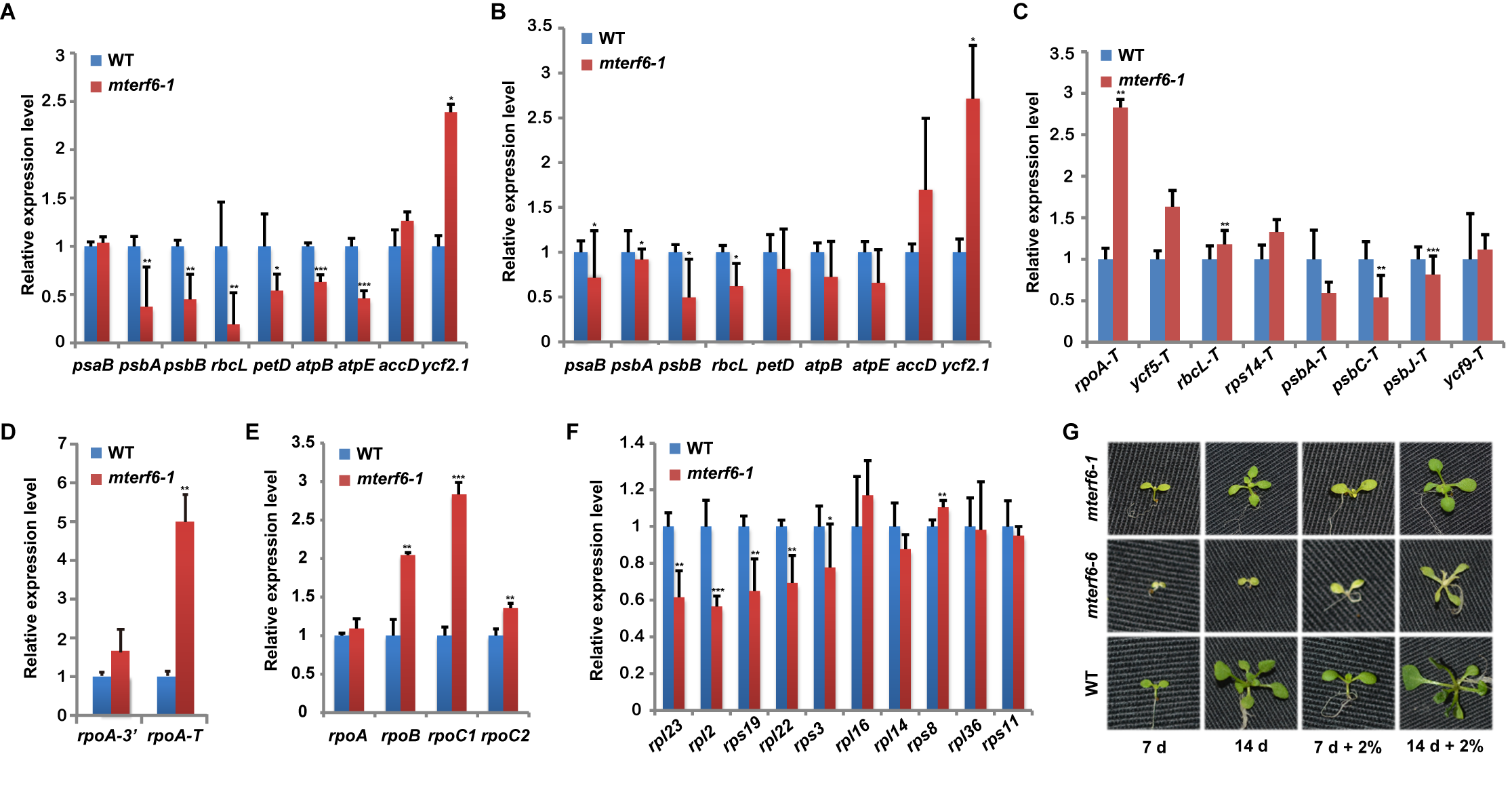


**Supplementary Figure 7. The functional study of mTERF6 in seedlings of *mterf6-1* mutant.**

**(A)** Quantitative real-time RT-PCR analysis of the three types of chloroplast genes in 7-day-old seedlings of WT and *mterf6-1*.

**(B)** Quantitative real-time RT-PCR analysis of the three types of chloroplast genes in 14-day-old seedlings of WT and *mterf6-1*.

**(C)** The read-through detection of selected genes by quantitative real-time RT-PCR.

**(D)** Quantitative real-time RT-PCR analysis of the *rpoA-3’* and *rpoA-T* transcripts in WT and *mterf6-1*. Primer pair *rpoA-3’* might be the 3’ end of precursor (Supplementary Fig. 4B). The less accumulated transcripts of *rpoA-3’* compared to that of *rpoA-T* suggests the read-through happens in the *mterf6-1* mutant.

**(E)** The gene expression of *rpoA*, *rpoB*, *rpoC1 and rpoC2* (encoding four core subunits of PEP) by quantitative real-time RT-PCR analysis.

**(F)** The gene expression of *L23-L2-S19-L22-S3-L16-L14-S8-L36-S11-rpoA* polycistron except *rpoA* by quantitative real-time RT-PCR analysis. In general, the reduced gene expression of *rpoA* polycistron suggests the transcription efficiency is decreased. The reduced expression of genes encoding ribosome subunits also hints translation efficiency may be reduced.

**(G)** The phenotypic characterization of *mterf6-1* compared to *mterf6-6* and WT. The *mterf6-1* mutant shows less severe growth retardation phenotype compared with the severe growth arrest phenotype of *mterf6-6* which show almost similar phenotype with *mterf6-5* on MS medium (Figure 1A).

Note: t-test for comparison between WT and *mterf6-1* mutants. “*”, “**”, and “***” indicate p<0.05, p<0.01, and p<0.001, respectively. Error bars indicate standard deviations for triplicates. Plants were grown on MS plates with/without 2% sucrose for 7 d or 14 d. For material used for quantitative real-time RT-PCR analysis, the plant growth condition of *mterf6-1* is the same as that of *mterf6-5*.


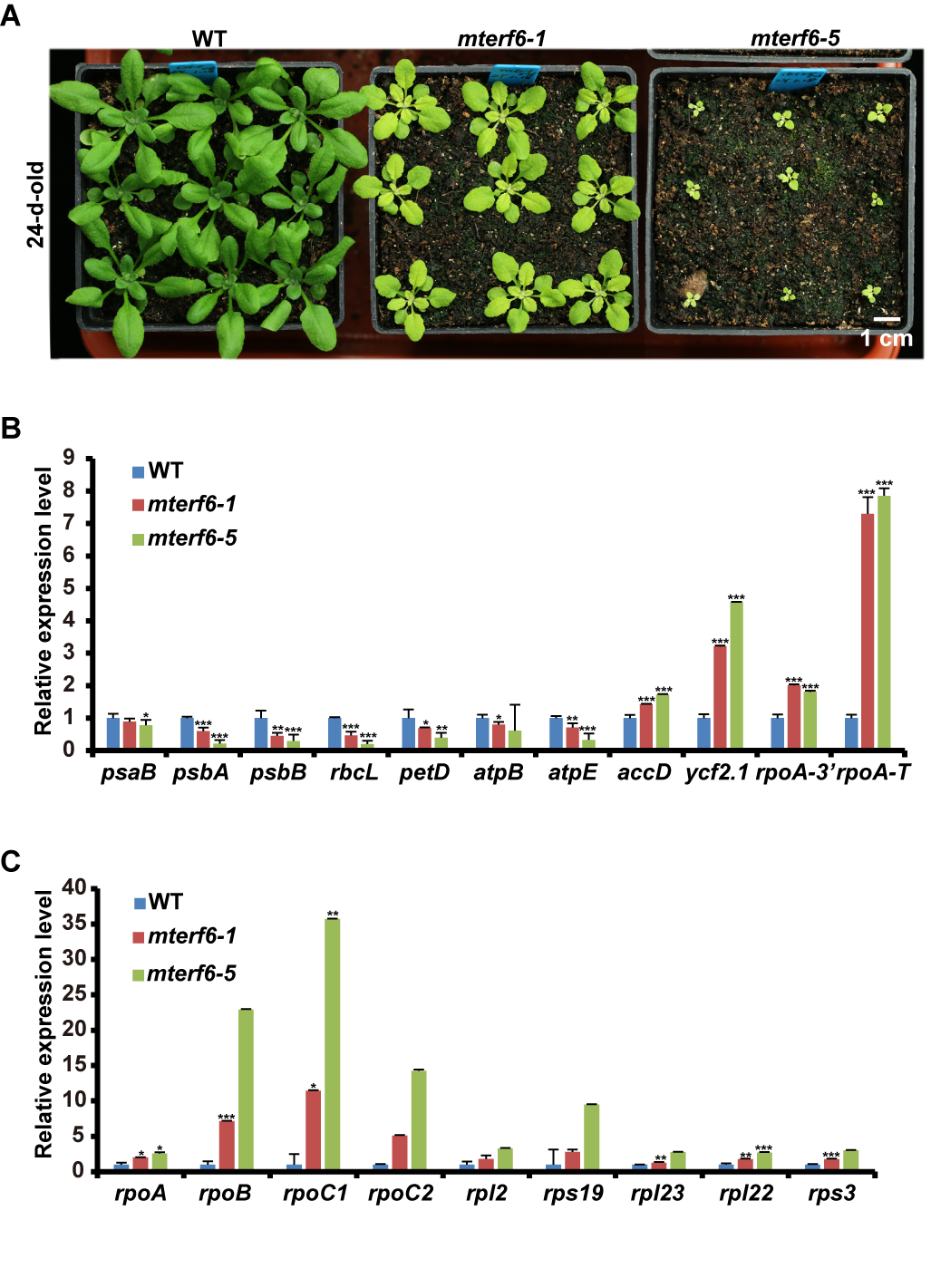


**Supplementary Figure 8. The functional study of mTERF6 in soil-grown *mterf6* mutants.**

**(A)** Phenotype characterization of the soil-grown WT and the *mterf6* mutants. Because the *mterf6-5* mutant is unable to survive after grown directly in soil for about 2 weeks, the WT, *mterf6-1* and *mterf6-5* were firstly grown on 2% sucrose containing MS plates for 5 days and then they were transplanted and kept growing in soil for another 19 days (total 24 days). Therefore, the 24-d-old plants were used as material for phenotypic observation and the following molecular function study of mTERF6. Bar, 1 cm.

**(B)** The detection of transcription levels of plastid genes and the *rpoA* read-through transcripts in the soil-grown WT and *mterf6* mutants by quantitative real-time RT-PCR.

**(C)** The detection of transcription levels of genes encoding PEP core subunits and some essential ribosomal subunits in the soil-grown WT and *mterf6* mutants by quantitative real-time RT-PCR.

Note: t-test for comparison between WT and the *mterf6* mutants. “*”, “**”, and “***” indicate p<0.05, p<0.01, and p<0.001, respectively. Error bars indicate standard deviations for triplicates.


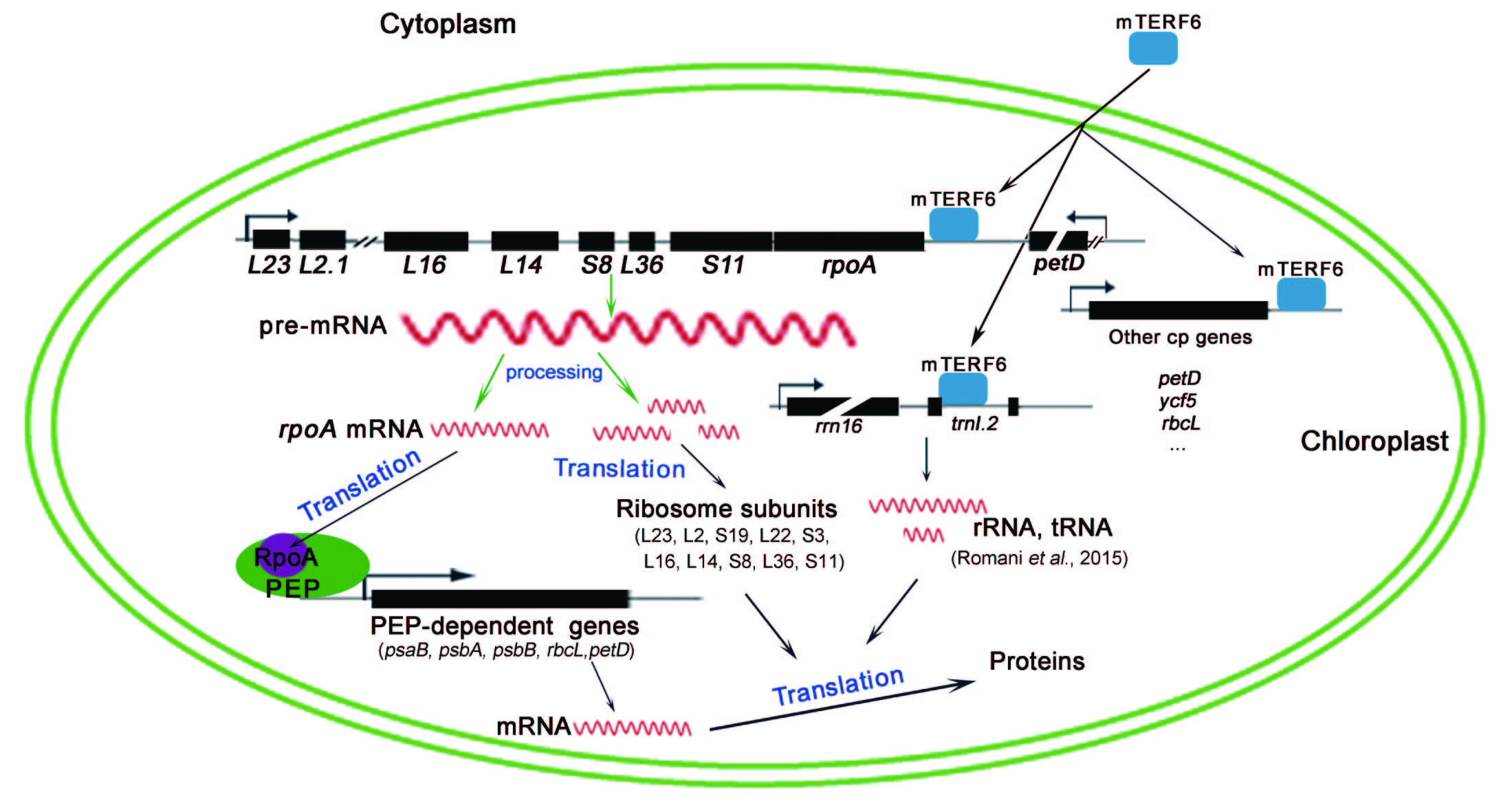


**Supplementary Figure 9.** A model for mTERF6 in the regulation of plastid gene expression.

mTERF6 directly binds to the 3’-end of *rpoA/petD* for transcription termination from two sides. The binding of mTERF6 mediates transcription efficiency of *L23-L2-S19-L22-S3-L16-L14-S8-L36-S11-rpoA* polycistron, which stabilizes, on the one hand, translation by adjusting transcription of essential ribosome subunits; on the other hand, PEP activity through controlling the RpoA protein level. The PEP regulates PEP-dependent plastid genes essential for chloroplast gene expression. Romani *et al*. (2015) demonstrated mTERF6 affects translation via binding to another site in *trnI.2* gene^37^. Together with our data, mTERF6 could also bind to other cp genes.

**Supplementary Table S1.** Primers used in this study.

| **Primer name** | **Primer sequence (5’-3’)** | | | **Primer usage** |
| --- | --- | --- | --- | --- |
|  | **Forward primer** | **Reverse primer** | |  |
| *LB1* | GCCTTTTCAGAAATGGATAAATAGC | | | genotyping |
| *LP* | ACTCTGCACAAGGCATCAACC | | | genotyping |
| *RP* | TGATGCCACATCGCTTTGAGC | | | genotyping |
| *SALK_116335LP* | ATTGTACCCTTGTCACGCATC | | | genotyping |
| *SALK_116335RP* | CGATGCTGTAGCTGATAAGCC | | | genotyping |
| *SacⅠ-MTERF6* | GAGCTCGTGGGGACGTTAGGAGAGTGACCAG | | | genetic complementation |
| *SalⅠ-MTERF6* | GTCGACTGATTACCTGCGATTATAGCCATTC | | | genetic complementation |
| *SalⅠ-MTERF6.1* | GTCGACGAAGCTTGCAGTTACCTCCGAAAAG | | | genetic complementation |
| *SalⅠ-MTERF6.2* | GTCGACAGCAGACACTTCTTTTGCTTGTCGG | | | genetic complementation |
| *SalⅠ-MTERF6.3* | GTCGACAATGTGACCTTTGACTAGGGGCCTC | | | genetic complementation |
| *MTERF6.1* | AGGTAACTGCAAGCTTCTAGC | | GAATAATCGATGATATCTGGA | RT-PCR, qRT-PCR |
| *MTERF6.2* | GTTGTTGGATAAGTCAGTGTC | | CATCTCTTTGTCATCAAATGT | RT-PCR, qRT-PCR |
| *MTERF6.3* | GTTGTTGGATAAAACAGAA | | CAAAAGAGAGAGCAGAGGG | RT-PCR, qRT-PCR |
| *TUB8* | GGACACTACACTGAAGGTGCTGAG | | CAAGCTGATGAACAGAGAGAGTTG | RT-PCR, qRT-PCR |
| *psaB* | GGACCCCACTACTCGTCGTA | | ATTGCTAATTGCCCGAAATG | qRT-PCR |
| *psbA* | GAGCAGCAATGAATGCGATA | | CCTATGGGGTCGCTTCTGTA | qRT-PCR |
| *psbB* | CGTGCGACTTTGAAATCTGA | | TAGCACCATGCCAAATGTGT | qRT-PCR |
| *rbcL* | GTGTTGGGTTCAAAGCTGGT | | CATCGGTCCACACAGTTGTC | qRT-PCR |
| *petD* | TCCTTTTGCAACTCCTTTGG | | CCGCTGGTACTGAAACCATT | qRT-PCR |
| *atpB* | CCGTTTCGTACAAGCAGGAT | | CGGGGTCAGTCAAATCATCT | qRT-PCR |
| *atpE* | TCCACAAGAAGCTCAGCAAA | | GTGTCCGAGCTCGTCTGAG | qRT-PCR |
| *accD* | TGTGGATTCAATGCGACAAT | | TTTTGCGCAGAGTCAATACG | qRT-PCR |
| *ycf2.1* | TAGCCCTCGGTCTATTGGTG | | GGATCCACTTTTTGGGGAAT | qRT-PCR |
| *rpoA* | GCGATGCGAAGAGCTTTACT | | CCAGGACCTTGGACACAAAT | qRT-PCR |
| *rpoB* | AAAAAGCACGGATACGGATG | | CTTCTTGAATGCCCCGATTA | qRT-PCR |
| *rpoC1* | TCGGATACGAAGATATCAAATGG | | TTAGTTATGGGCCTAGCAAAAGA | qRT-PCR |
| *rpoC2* | ATGGAGCCCGTAAAGGAGTT | | CGTCTGCTAAGACACGACCA | qRT-PCR |
| *rpL23* | CGGTTATTGGGGAAAAATCA | | TTTTAACCTTTCCGGGGAGT | qRT-PCR |
| *rpL2* | CGGACCTCTCCAGAAGGTAAT | | AAATGGGAAATGCCCTACCT | qRT-PCR |
| *rpS19* | CACAATGATTGGCCATACGA | | TTTGGCATGTCCTCGAAAAT | qRT-PCR |
| *rpL22* | AAAGCTGAGGTGAACCAAGG | | TGTCCCATAGGCCTCCACTA | qRT-PCR |
| *rpS3* | CAATCCGTATGGGGATCCTA | | GATCCATTCAACACGTGCAA | qRT-PCR |
| *rpL16* | TGTACGACGTGGTGGAAAAA | | GCATTTTTGATGCCGCTATT | qRT-PCR |
| *rpL14* | AGCGGGGCTAGAGAATTGAT | | ACTGCGGCATTGTCATCATA | qRT-PCR |
| *rpS8* | CGACCGGGTCTACGAATCTA | | ATTTCTCCGCCGATTCTTTT | qRT-PCR |
| *rpL36* | AAATAAGGGCTTCCGTTCGT | | CCTCGGGTTGGAACAAATTA | qRT-PCR |
| *rpS11* | TACTTGTGGATTCCGGGGTA | | CAGCTCGTTGCATACCTTGA | qRT-PCR |
| *psbJ* | ATGGCTGATACTACTGGAAGGA | | GGGATGAACCTAATCCTGAATA | qRT-PCR |
| *psbJ-T* | TCAAGAAGGACAAGATCGACACA | | AAATAAGATAAGGAACACACGCG | qRT-PCR, ChIP-qPCR |
| *rbcL-T* | AAAAAAAGAAGATTAAACACAACT | | AATACGGAATGAAAAGAAAAGGAC | qRT-PCR, ChIP-qPCR |
| *ycf9* | TTACTGATTAGTGTACCCGTT | | AATTAAGGATACCCACCAAGA | qRT-PCR |
| *ycf9-1* | GTCTTCTTGGTGGGTATCC | | CCCCCCTCTAATTTTTTCT | qRT-PCR |
| *ycf9-2* | AGATGACCCCCTCCCATTC | | TTCCATAAGTTCGACCCCC | qRT-PCR |
| *ycf9-T* | TCTGGAATTCAATATTAAATTA | | AAAGCTAGACGAGATACACTCT | qRT-PCR, ChIP-qPCR |
| *ycf5* | CATTAATTTTTCTTTCGTGGGC | | ACTGGGACTGGAGAGCTGGTAC | qRT-PCR |
| *ycf5-T* | ATAAAAAAATAAAAAAATAGCATC | | TTAAAGTGAATTGTAATCAGAAGT | qRT-PCR, ChIP-qPCR |
| *psbC* | CCCTTTTTCGGTTATGTATGG | | TTGGGCTAAGAGTCAAGTTTG | qRT-PCR |
| *psbC-T* | AGTAGGTAAAACAAATAGGTTCATA | | ATCTATTTTTCTCTTTATTGGCTT | qRT-PCR, ChIP-qPCR |
| *rps14* | ATAGGGAGAAGAAGAGGCAAA | | AACGTCGATGAAGACGTGTAG | qRT-PCR |
| *rps14-T* | CTGAAACAAAAAAACTTGGATT | | GGATTTAAGTATCCCTTAATTT | qRT-PCR, ChIP-qPCR |
| *petD-T* | CCTGTGGTTAGGTATTGGAGCA | | ATCTAGGGAGAATTCATTTGGA | qRT-PCR, ChIP-qPCR |
| *psbA-T* | TATGGAAGTTATGCATGAACGTA | | CCTCTTTCTTATTTAAAGAAGGC | qRT-PCR, ChIP-qPCR |
| *trnI.2-WT* | GATAATTGCGTCGTTGTGCCTGGA | | CTATTAACGCAGCAACACGGACCT | EMSA |
| *trnI.2-M1* | GATACCCGCGTCGTTGTGCCTGGA | | CTATTAACGCAGCAACACGGACCT | EMSA |
| *trnI.2-M2* | GATAATTGCGTCGTTACGCCTGGA | | CTATTAACGCAGCAACACGGACCT | EMSA |
| *probe A* | AATTTCTATTTTCCAGTCGAAC | | GTGGGTATCTAGGGAGGTATCT | ChIP-qPCR, EMSA |
| *probe B* | AATTTCTATTTTCCAGTCGAAC | | GTTCGACTGGAAAATAGAAATT | ChIP-qPCR, EMSA |
| *RB7* | ATTATTTCGTGGATTGGATTTCA | | TTACGCTTCAACCGGGTTATTC | ChIP-qPCR, EMSA |
| *mTERF6.1-pMAL-c5x* | GGGAATTCCATATGATGAAGATGTTGAGAAAATGCAAAC | | CGGGATCCCTAGAAGCTTGCAGTTACCTCCGAA | Protein expression |
| *rpl2* | ACACTATGGGGATGGTGAGAA | | TCTTTTGCAATCAGTTTCGC | RT-PCR (splicing detection) |
| *rpl16* | GATATAATTGCTATGCTTAGTG | | GAAGAGCATATCTGCCAAAAC | RT-PCR (splicing detection) |

**Cited References:**

37. Romani, I. *et al*. mTERF6, a member of the *Arabidopsis* mitochondrial transcription termination factor family, is required for maturation of chloroplast tRNA^Ile (GAU)^. *Plant Physiol.* **169**, 627-646 (2015).

44. Chateigner-Boutin, A.L. *et al*. CLB19, a pentatricopeptide repeat protein required for editing of *rpoA* and *clpP* chloroplast transcripts. *Plant J.* **56**, 590-602 (2008).

48. Roberti, M., Mustich, A., Gadaleta, M.N., & Cantatore, P. Identification of two homologous mitochondrial DNA sequences, which bind strongly and specifically to a mitochondrial protein of *Paracentrotus lividus*. *Nucleic Acids Res.* **19**, 6249-6254 (1991).

50. Wobbe, L. & Peter, J.N. The mTERF protein MOC1 terminates mitochondrial DNA transcription in the unicellular green alga *Chlamydomonas reinhardtii*. *Nucleic Acids Research* **10**, 1-15 (2013).

58. Roberti, M. *et al*. DmTTF, a novel mitochondrial transcription termination factor that recognises two sequences of Drosophila melanogaster mitochondrial DNA. *Nucleic Acids Res.* **31**, 1597-1604 (2003).

75. Christianson, T.W. & Clayton, D.A. In vitro transcription of human mitochondrial DNA: accurate termination requires a region of DNA sequence that can function bidirectionally. *Proc. Natl. Acad. Sci. USA* **83**, 6277-6281 (1986).

**Original pictures of gels and blots**


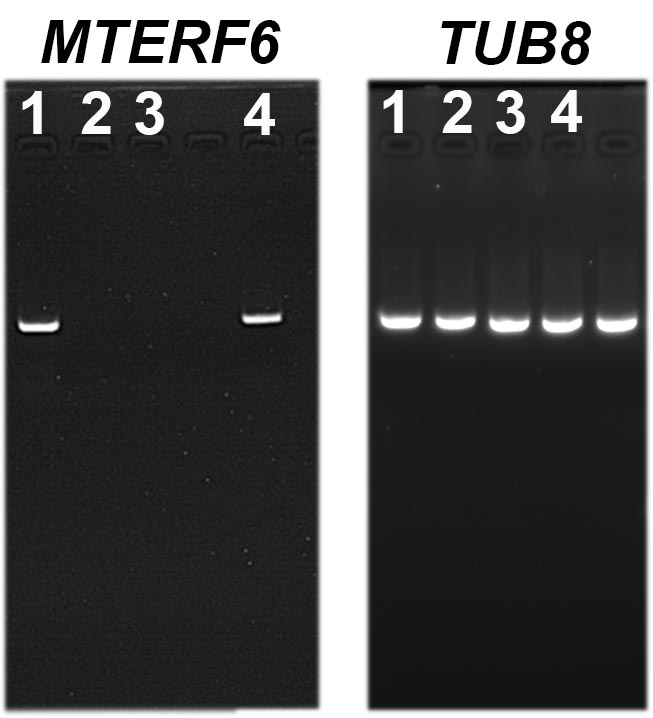


**Original pictures of Figure 1D.**

Line 1, 2, 3 and 4 represent transcript levels in WT, *mterf6-5*, *mterf6-6*, and *mterf6-5* complemented seedlings, respectively. Left picture shows the detected *MTERF6* transcripts, while the right picture indicates the *TUB8* transcripts as *a* control.


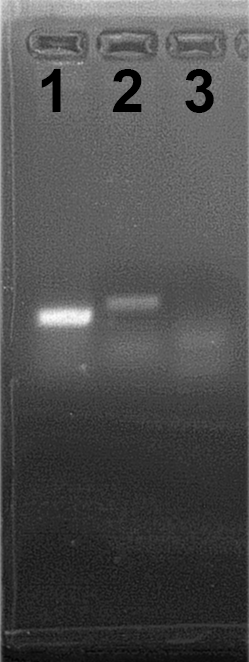


**Original picture of Figure 2B.**

Line 1, 2 and 3 indicate the expression levels of *AT4G38160.1*, *AT4G38160.2*, and *AT4G38160.3* in seedlings, respectively.


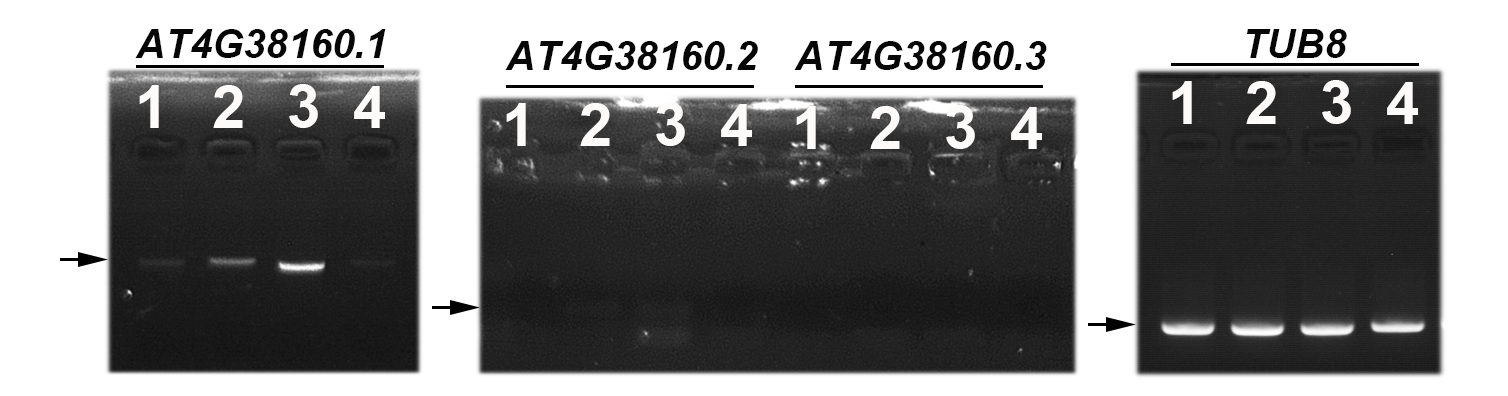


**Original picture of Figure 2C.**

Line 1, 2, 3 and 4 denote the gene expression levels in root, stem, leaf and flower tissues, respectively. Arrows denote the detected gene expression levels of *AT4G38160.1*, *AT4G38160.2*, *AT4G38160.3* and *TUB8* in these different tissues by RT-PCR.

**
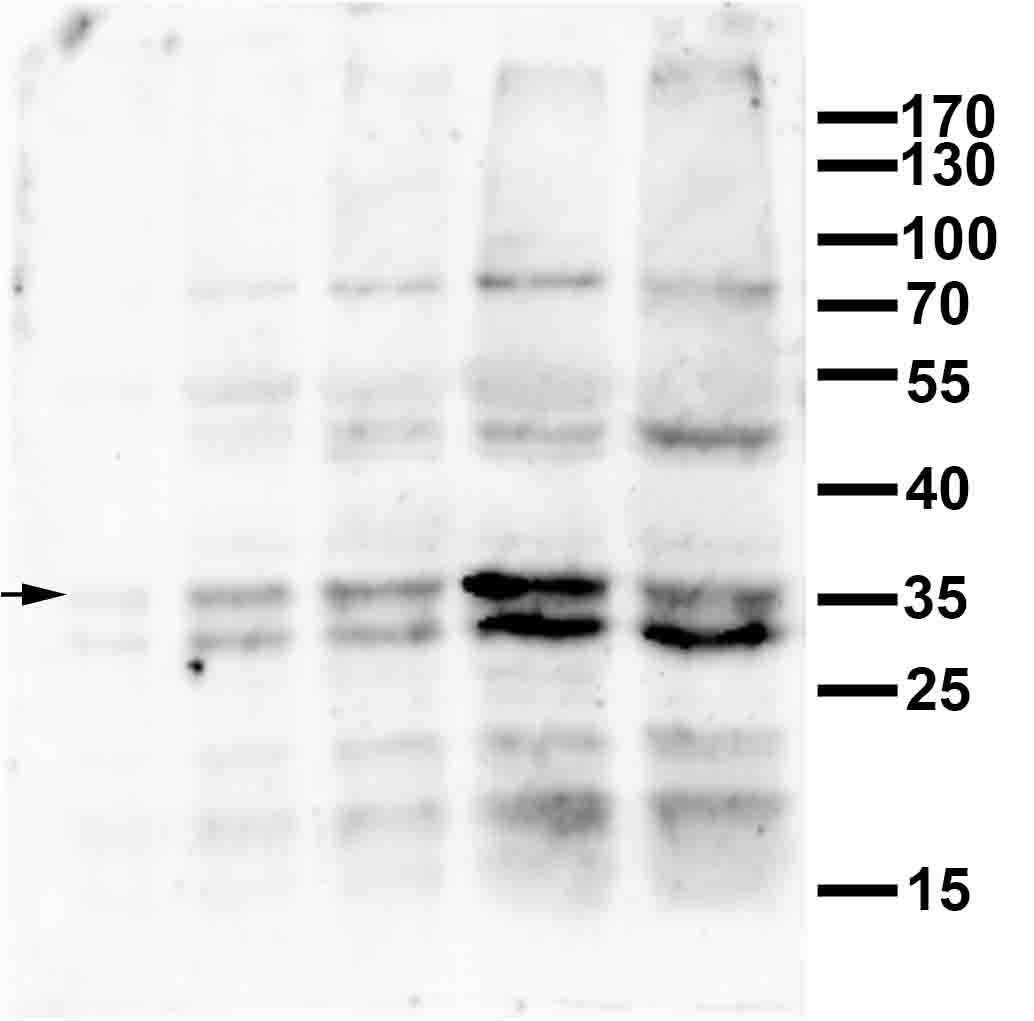
**

**Original picture of Figure 3B (top panel).**

A band with a molecular mass of ~38 kDa, corresponding to the predicted molecular weight of RpoA protein, is indicated by an arrow on the left. Protein molecular size marker (kDa) is depicted on the right.


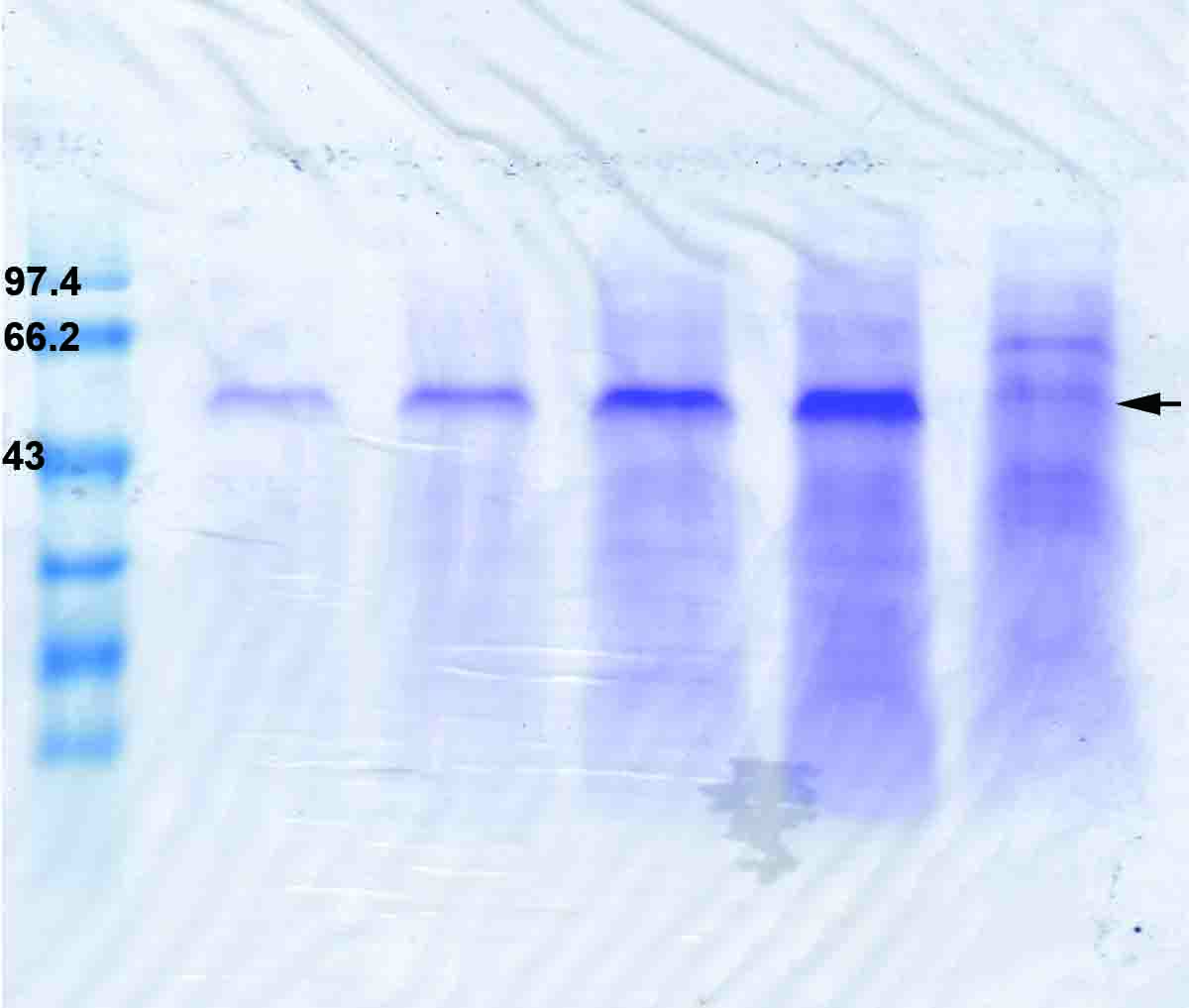


**Original picture of Figure 3B (bottom panel).**

Protein molecular size marker (kDa) is depicted on the right. The position of RbcL is indicated by an arrow on the right.


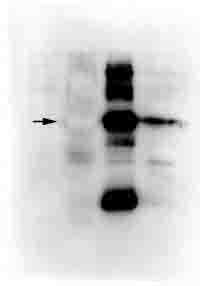


**Original picture of Figure 5A.**

This picture indicates the full-length blot of mTERF6 immunoprecipitation by using anti-FLAG antibody (Sigma). The ~40 kDa band is the target mTERF6-FLAG protein extracted from *pMTERF6::MTERF6.1-FLAG* transgenic plants indicated by an arrow on the left. Three lanes from left to right indicate the control, the anti-FLAG immunoprecipitated products, and the input, respectively.


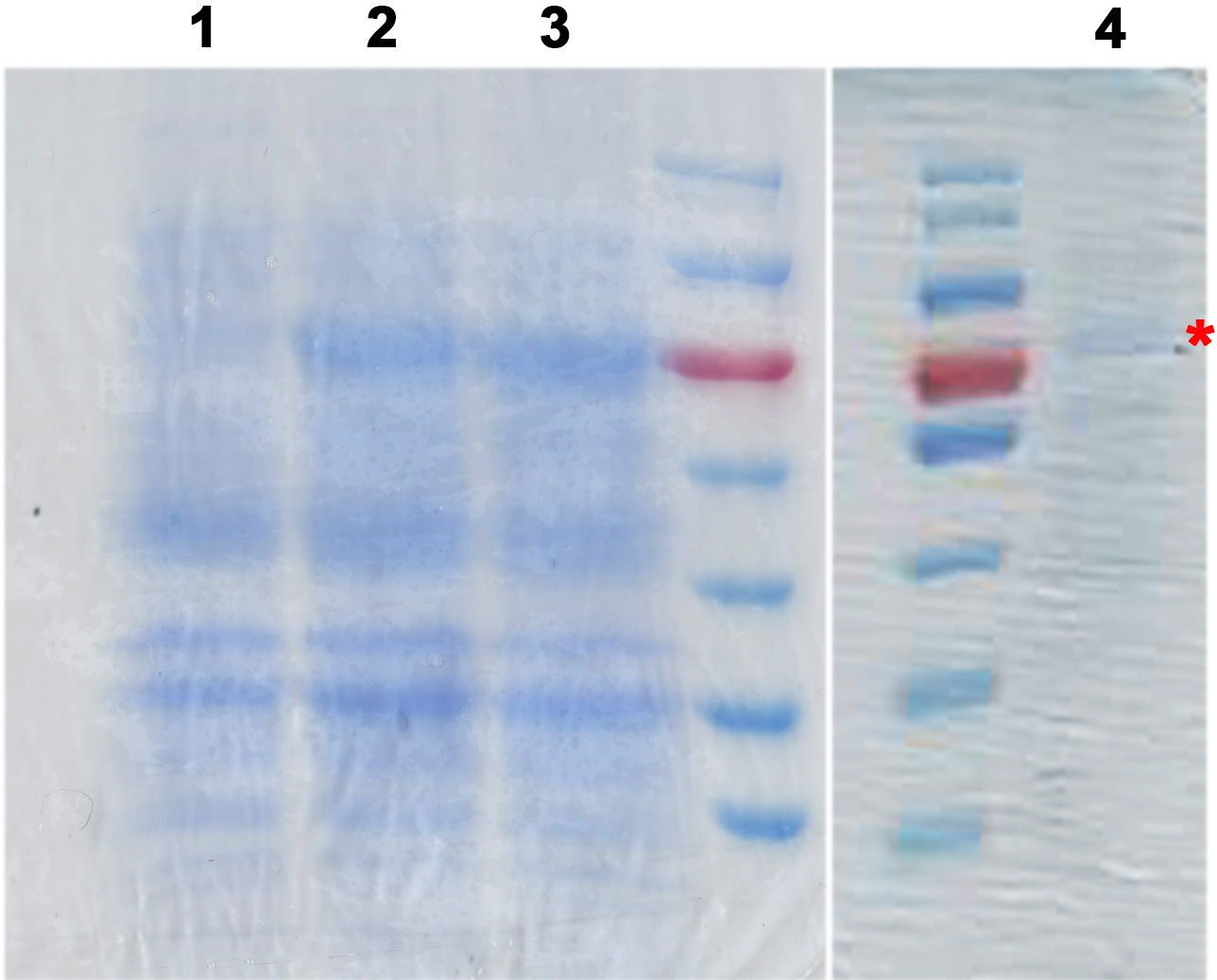


**Original pictures of Figure 6B.**

1, 2, 3, and 4 denote lane 1, lane 2, lane 3 and lane 4, respectively, corresponding to that in Figure 6B.


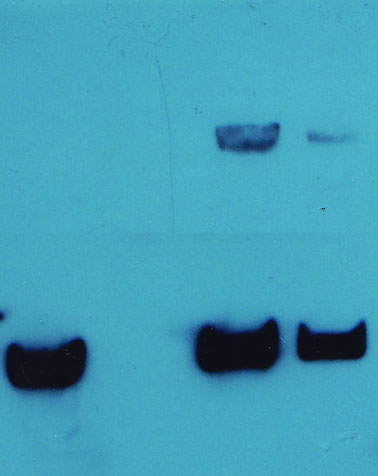


**Original picture of Figure 6C.**

Four lanes from left to right denote free probe, free protein, probe plus protein, and probe plus protein and competitor, respectively.

**
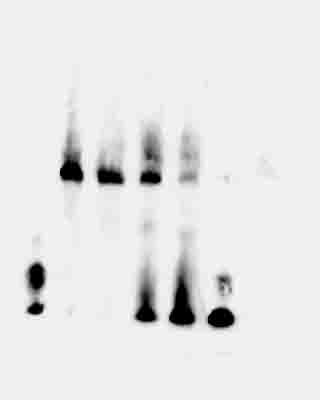
**

**Original picture of Figure 6D.**

7 lanes from left to right are in correspondence with lane 1 to 7 of Figure 6D.


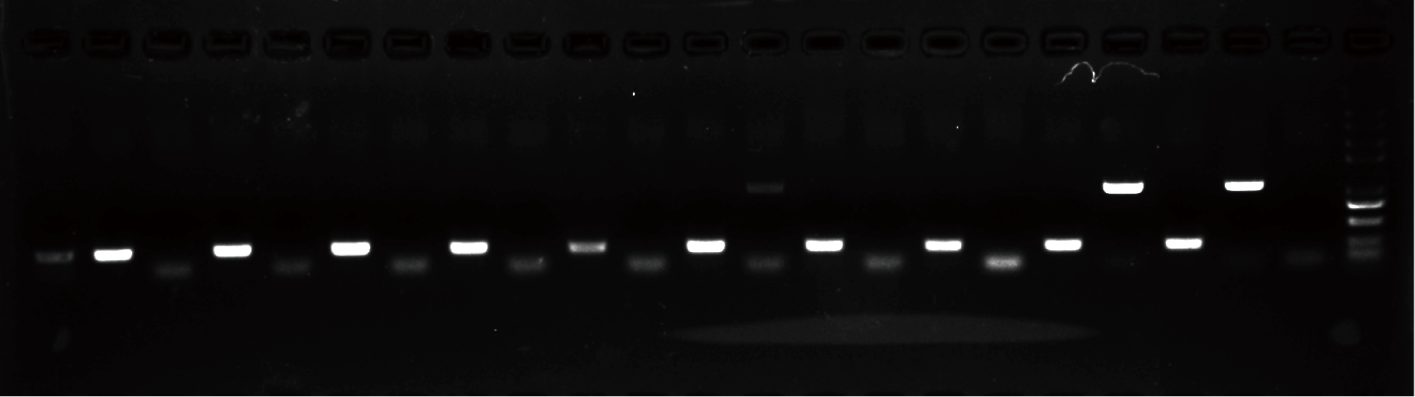


**Original pictures of Supplementary Figure 1C (middle panel).**


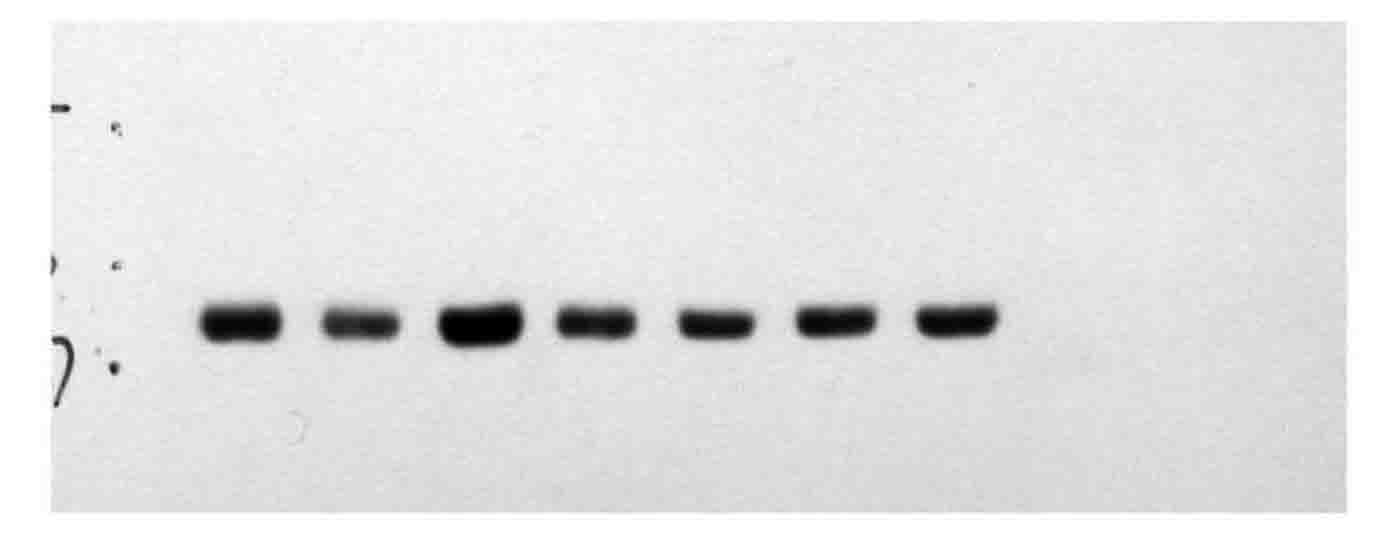


**
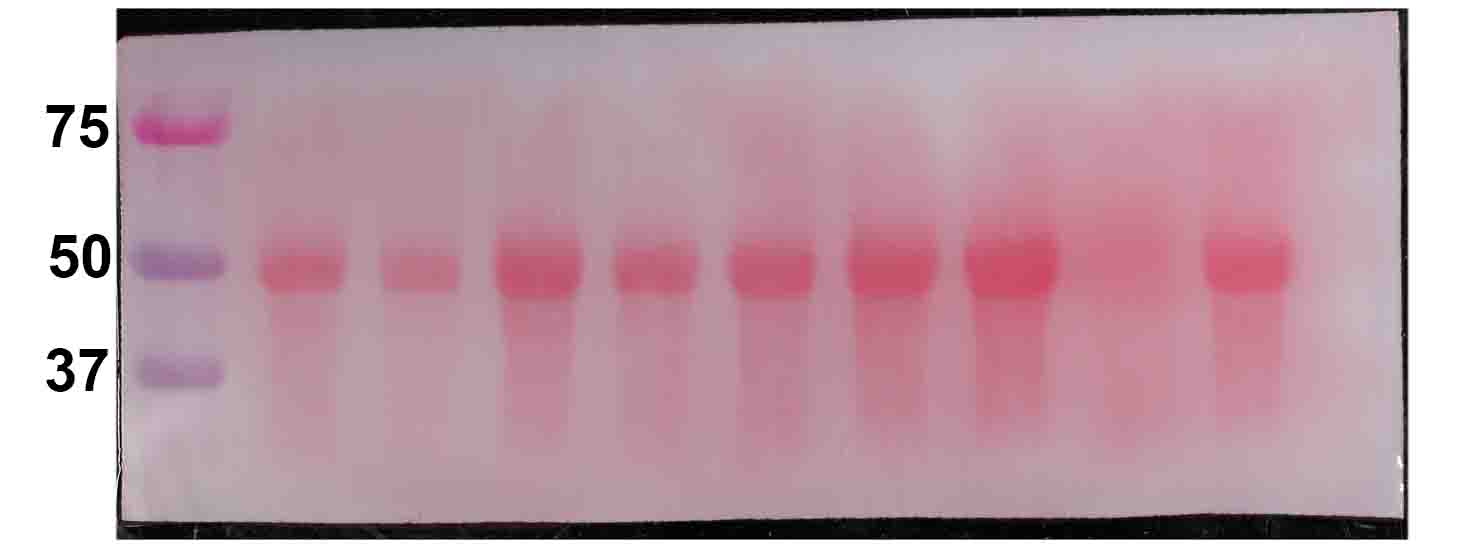
**

**Original pictures of Supplementary Figure 1C (bottom panel).**

Top panel shows the results of western blot detected by anti-FLAG antibody (Sigma). Bottom panel indicates the Ponceau S staining of total proteins. Protein molecular size marker (kDa) is depicted on the left.


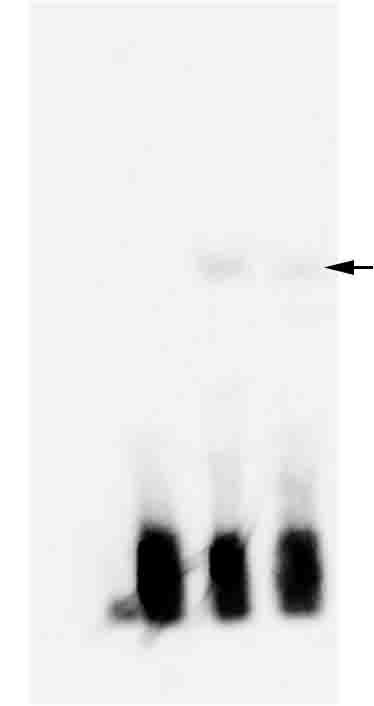


**Original pictures of Supplementary Figure 4C (left panel).**

4 lanes from left to right are in correspondence with that in Supplementary Figure 6D (left panel). An arrow on the right indicates the position of the shifted band.

**
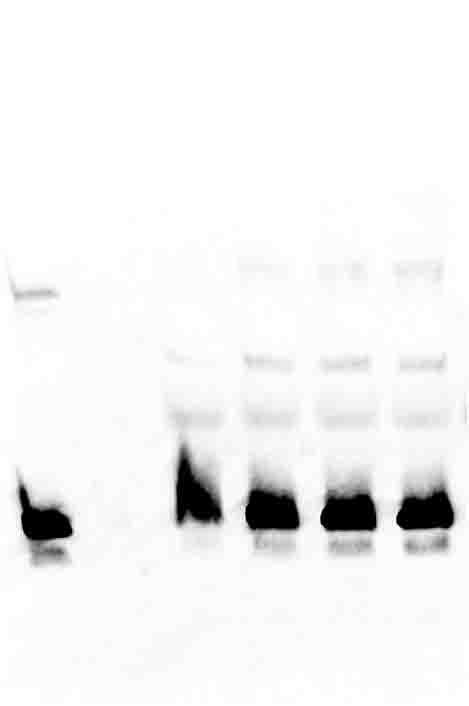
**

**Original pictures of Supplementary Figure 4C (middle panel).**

6 lanes from left to right are in correspondence with that in Supplementary Figure 4C (middle panel).

**
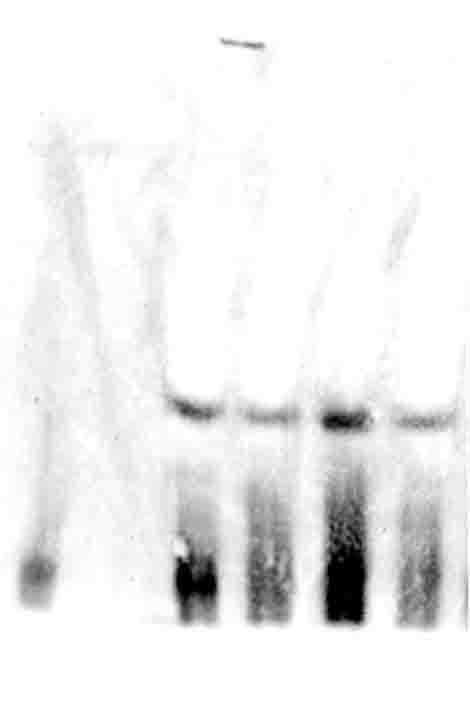
**

**Original pictures of Supplementary Figure 4C (right panel).**

6 lanes from left to right are in correspondence with that in Supplementary Figure 4C (right panel).


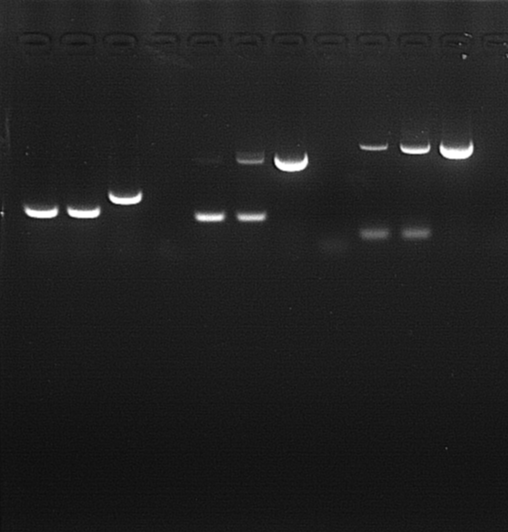


**Original pictures of Supplementary Figure 6E.**
